# Genotyping‐by‐sequencing and ecological niche modeling illuminate phylogeography, admixture, and Pleistocene range dynamics in quaking aspen (*Populus tremuloides*)

**DOI:** 10.1002/ece3.6214

**Published:** 2020-04-23

**Authors:** Justin C. Bagley, Neander M. Heming, Eliécer E. Gutiérrez, Upendra K. Devisetty, Karen E. Mock, Andrew J. Eckert, Steven H. Strauss

**Affiliations:** ^1^ Plant Evolutionary Genomics Laboratory Department of Biology Virginia Commonwealth University Richmond VA USA; ^2^ Departamento de Zoologia Instituto de Ciências Biológicas Universidade de Brasília Brasília Brazil; ^3^ Programa de Pos‐Graduação em Biodiversidade Animal Centro de Ciências Naturais e Exatas Universidade Federal de Santa Maria Santa Maria Brazil; ^4^ CyVerse Bio5 University of Arizona Keating Bioresearch Building Tucson AZ USA; ^5^ Department of Wildland Resources and Ecology Center Utah State University Logan UT USA; ^6^ Department of Forest Ecosystems and Society Oregon State University Corvallis OR USA

**Keywords:** admixture, North America, phylogeography, population structure, quaking aspen, single nucleotide polymorphisms

## Abstract

*Populus tremuloides* is the widest‐ranging tree species in North America and an ecologically important component of mesic forest ecosystems displaced by the Pleistocene glaciations. Using phylogeographic analyses of genome‐wide SNPs (34,796 SNPs, 183 individuals) and ecological niche modeling, we inferred population structure, ploidy levels, admixture, and Pleistocene range dynamics of *P. tremuloides*, and tested several historical biogeographical hypotheses. We found three genetic lineages located mainly in coastal–Cascades (cluster 1), east‐slope Cascades–Sierra Nevadas–Northern Rockies (cluster 2), and U.S. Rocky Mountains through southern Canadian (cluster 3) regions of the *P. tremuloides* range, with tree graph relationships of the form ((cluster 1, cluster 2), cluster 3). Populations consisted mainly of diploids (86%) but also small numbers of triploids (12%) and tetraploids (1%), and ploidy did not adversely affect our genetic inferences. The main vector of admixture was from cluster 3 into cluster 2, with the admixture zone trending northwest through the Rocky Mountains along a recognized phenotypic cline (Utah to Idaho). Clusters 1 and 2 provided strong support for the “stable‐edge hypothesis” that unglaciated southwestern populations persisted in situ since the last glaciation. By contrast, despite a lack of clinal genetic variation, cluster 3 exhibited “trailing‐edge” dynamics from niche suitability predictions signifying complete northward postglacial expansion. Results were also consistent with the “inland dispersal hypothesis” predicting postglacial assembly of Pacific Northwestern forest ecosystems, but rejected the hypothesis that Pacific‐coastal populations were colonized during outburst flooding from glacial Lake Missoula. Overall, congruent patterns between our phylogeographic and ecological niche modeling results and fossil pollen data demonstrate complex mixtures of stable‐edge, refugial locations, and postglacial expansion within *P. tremuloides*. These findings confirm and refine previous genetic studies, while strongly supporting a distinct Pacific‐coastal genetic lineage of quaking aspen.

## Graphical abstract

We inferred the phylogeographic history of *Populus tremuloides* by combining broadscale inferences of population structure, admixture, and ploidy based on genome‐wide SNP data with spatially explicit predictions of the past to present geographical distributions of the species and its intraspecific lineages (genetic “clusters”) using ecological niche modeling (ENM) hindcasting. Under this framework, we found strong evidence for significant patterns of population divergence and admixture among three intraspecific genetic clusters, including a new and genetically distinct lineage of Pacific‐coastal aspen. Our results from integrating these approaches to analyze intraspecific genetic clusters within *P. tremuloides* also obtained strong support for stable‐edge dynamics, but mixed support for trailing‐edge dynamics. Overall, our findings agree well with the previous genetic results but present a more nuanced picture of *P. tremuloides* evolution and diversification refining the geographical positions of genetic subdivisions, past or ongoing admixture, and putative Pleistocene refugia.

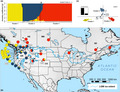

## 1 INTRODUCTION

Many North American forest tree species are broadly distributed across large areas of the continent (Little, 1971; Prasad, Iverson, Matthews, & Peters, 2007). Closely related populations and species frequently occur across major geographical barriers, including western Pacific mountain ranges (e.g., Cascades, Sierra Nevada), ranges of the greater continental divide (Rocky Mountains) and eastern divide (Appalachian Mountains), or xeric habitats of the Great Basin and major deserts. Explaining such distributions requires historical biogeographical processes such as range fragmentation in a wide‐ranging ancestral lineage spanning both sides of a barrier, or dispersal into areas on either side (e.g., Rosen, 1978). Pleistocene glaciations represent another major factor influencing the distributions and genetic diversity of North American tree species (Hewitt, 1996, 2001; Jaramillo‐Correa, Beaulieu, Khasa, & Bousquet, 2009; Soltis, Morris, McLachlan, Manos, & Soltis, 2006). Fossil pollen and plant macrofossil data show that the advance and retreat of massive continental ice sheets during the mid‐late Pleistocene cyclically reduced population sizes and forced extirpations of forest trees from northern glaciated areas, followed by predominantly northward population/range expansion during postglacial recolonization (Jackson et al., 2000; Williams, Shuman, Webb, Bartlein, & Leduc, 2004; but see Provan & Bennett, 2008). Teasing apart these historical and evolutionary processes in a geographical context to explain the diversity, demography, and assembly of North American tree communities has been a key goal of phylogeography for >20 years (Sewell, Parks, & Chase*,* 1996; Soltis, Gitzendanner, Strenge, & Soltis, 1997; Mitton, Kreiser, & Latta, 2000; Cheddadi et al., 2006; Fazekas & Yeh, 2006; Godbout, Fazekas, Newton, Yeh, & Bousquet, 2008; O’Connell, Ritland, & Thompson, 2008; Eckert et al., 2010; Gugger, González‐Rodríguez, Rodríguez‐Correa, Sugita, & Cavender‐Bares, 2010; Keller, Olson, Silim, Schroeder, & Tiffin, 2010; Breen, Murray, & Olson, 2012).

**Figure 1:**
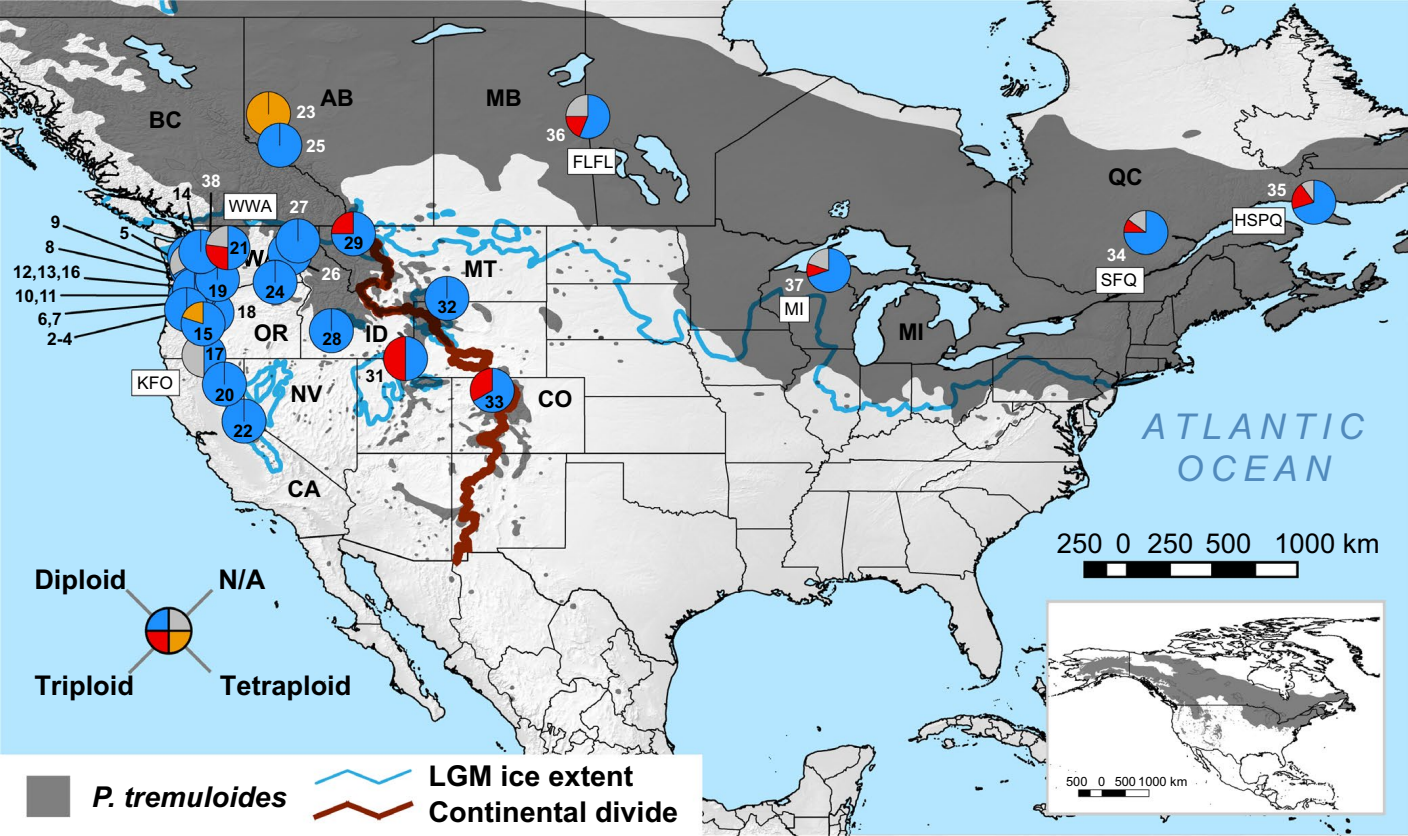
Sampling sites and ploidy proportions within *Populus tremuloides* subpopulations. Pie charts show the proportion of samples inferred to be diploid, triploid, or tetraploid in nQuire (Weiß et al., 2018), which uses Gaussian mixture models (GMMs) and ML approaches to model biallelic SNP frequencies across Illumina reads from each individual, mapped to the *P. tremuloides* reference genome. Gray pie proportions (labeled “N/A”) represent individuals whose cytotypes were not assigned because they did not meet nQuire's allele frequency or read coverage cutoffs. Results are mapped over a topographic map of the study area showing the *P. tremuloides* geographical range from Little (1971; dark gray shading), the extent of ice sheets during the Last Glacial Maximum (LGM; blue line), and the position of continental divide (thick dark red line). Inset: full species range (Little, 1971) extending into Alaska and northern Canada

Quaking aspen, *Populus tremuloides* Michx., is the most widely distributed North American tree species, ranging from northern Canada southward into pockets of central Mexico (Figure 1; Little, 1971). While common above 500 m elevation, *P. tremuloides* occur in distinct microhabitats throughout their range, with montane western populations occurring in riparian corridor, treeline, and krümmholz stands (Shepperd, Rogers, Burton, & Bartos, 2006) and eastern populations more likely encountered along rivers and riparian zones (Barnes & Wagner, 2002). In addition to striking morphological variation (Barnes, 1967, 1975), *P. tremuloides* possess among the highest genetic diversity reported for *Populus* species to date (e.g., Callahan et al., 2013; Cole, 2005; Jelinski & Cheliak, 1992; Wang, Street, Scofield, & Ingvarsson, 2016), and genetic resources include linkage maps and a draft annotated genome (Pakull, Groppe, Meyer, Markussen, & Fladung, 2009; Sjödin, Street, Sandberg, Gustafsson, & Jansson, 2009; Sundell et al., 2015). These qualities make quaking aspen an ideal system for studying the genomic and ecological contexts of speciation and local adaptation in forest trees. Historically, unglaciated portions of the species range correspond to major glacial‐stage refugia in the Cascades/unglaciated Northern Rocky Mountains and the mid‐southern Rocky Mountain Front, based on phylogeographic data from many plant and animal taxa (reviewed in Brunsfeld, Sullivan, Soltis, & Soltis, 2001; Jaramillo‐Correa et al., 2009). Therefore, *P. tremuloides* also presents outstanding opportunities for testing historical biogeographical hypotheses on the locations of forest tree refugia and the formation of mesic, temperate forest ecosystems of the Pacific Northwest and Rocky Mountains.

Several important gaps in our knowledge of *P. tremuloides* evolutionary history remain open to investigation, including whether additional data from high‐throughput sequencing might confirm or refine previously described intraspecific genetic clusters (Callahan et al., 2013), and the nature of Pleistocene range dynamics [e.g., refugia since the Last Interglacial (LIG), but see Ding, Schreiber, Roberts, Hamann, and Brouard (2017)] and their influence on intraspecific genetic variation. To address these knowledge gaps, we investigate the genetic structure and population history of *P. tremuloides* by integrating phylogeographical analyses of genotyping‐by‐sequencing (GBS) data (Elshire et al., 2011) with ecological niche modeling (ENM; Peterson et al., 2011) analyses predicting the species Pleistocene to recent geographical range dynamics. Using a high ratio of genome‐wide SNPs to individuals is an optimal sampling design (e.g., Felsenstein, 2006) that has been shown to yield high‐resolution inferences of population history, even with small–moderate sample sizes (e.g., Gutenkunst, Hernandez, Williamson, & Bustamante, 2009; Willing, Dreyer, & Oosterhout, 2012; Robinson, Bunnefeld, Hearn, Stone, & Hickerson, 2014; Boehm, Waldman, Robinson, & Hickerson, 2015). Thus, we sought a balance between numerical and genomic sampling by offsetting losses of information from sampling on average ~5 (median: 3; range: 1–22) individuals per local subpopulation (=site) with thousands of unlinked single nucleotide polymorphism (SNP) loci from throughout the genome. Our specific goals were fourfold: (a) to infer broadscale patterns of population structuring and ploidy levels within *P. tremuloides* using genomic SNP data; (b) to test the presence and directionality of admixture between intraspecific gene pools; (c) to infer Pleistocene range dynamics of the species and its genetic lineages using ENMs and assess whether genetic differentiation is explained by connectivity or isolation of predicted suitable habitats over the last glacial cycle; and (d) to test several a priori historical biogeographical hypotheses for North American forest trees described below.

## 2 MATERIALS AND METHODS

### 2.1 Historical biogeographical hypotheses

Our study design permitted testing two pairs of competing a priori historical biogeographical hypotheses and one stand‐alone hypothesis. First, we expected (H_1_) the “stable‐edge hypothesis” of long‐term persistence of *P. tremuloides* populations in the intermountain west to be strongly supported if predicted suitable areas were fully or partly stable from the LGM to present, had higher genetic or phylogeographic diversity (number of lineages), and exhibited significant isolation‐by‐geographical distance (Callahan et al., 2013; Hampe & Petit, 2005). The opposite (H_2_) “trailing‐edge hypothesis” would be supported if predicted suitable areas were completely latitudinally displaced during the LGM and populations exhibited clinal genetic patterns indicating large‐scale postglacial population expansions or lower phylogeographic diversity (e.g., Excoffier, Foll, & Petit, 2009; Hampe & Petit, 2005; Hewitt, 1996, 2001). Based on previous studies, we expected stable‐edge dynamics in “southwestern” cluster populations and trailing‐edge dynamics in “northern cluster” populations of *P. tremuloides* (Callahan et al., 2013). *Populus tremuloides* also exhibits the “mesic forest disjunct pattern” of Brunsfeld et al., (2001), with Pacific‐coastal populations separated from interior Rocky Mountain populations by intervening arid habitats of the Columbia Plateau in east‐central Washington. Thus, we used our genetic results to test two nonmutually exclusive regional biogeographical hypotheses proposed to explain this distributional pattern. We tested (H_3_) the well‐known “ancient vicariance hypothesis” positing that Cascade/Costal range forests became isolated from Northern Rocky Mountain forests during the Pliocene formation of the Cascades Range (Daubenmire, 1975) against (H_4_) the “inland dispersal hypothesis” that mesic forests recolonized the Northern Rockies since the LGM without substantial genetic divergence from coastal populations (Brunsfeld et al., 2001; Brunsfeld & Sullivan, 2005; Cartens, Brunsfeld, Demboski, Good, & Sullivan, 2005). During postglacial retreat of continental glaciers, large lakes formed and eventually overflowed, flooding to coastlines and sparking extirpations or dispersals of northern populations of plants and animals from the Pacific Northwest (Pielou, 1991). Among the largest, glacial Lake Missoula in western Montana repeatedly flooded the eastern Washington scablands and inundated the Willamette Valley in Oregon and the Columbia Basin to the Pacific coast, with the most massive events occurring since ~20 ka, and most recently ~14.7 ka (Balbas et al., 2017, refs. therein). Thus, we tested the “Missoula floods hypothesis” that Pacific‐coastal *P. tremuloides* populations were colonized by transfer of propagules during these latest pronounced Missoula floods, which would predict that at least some populations in coastal Oregon and Idaho–Montana exhibit genetic similarity.

### 2.2 Sampling and laboratory methods for genomic data

We obtained samples of leaves or stem cuttings from 96 *P. tremuloides* trees from 33 local sites (1–5 trees per site) across the species native range in western North America (Figure 1 and Figure S1), including samples from 11 sites in Callahan et al. (2013). Additional information on sampling site locations and tissue sources of individual trees is provided in Data S1 of the Supporting Information. We extracted genomic DNA from tissues using an in‐house, phenol–chloroform DNA extraction protocol available in our Mendeley Data accession. Presence and quantity of extracted DNA was quantified using a NanoDrop ND‐1000 spectrophotometer (Thermo Fisher Scientific), and DNA quality was assessed by electrophoresis on 1% agarose gels. DNA concentrations were normalized before a GBS library was prepared containing 96 multiplexed samples according to the genotyping‐by‐sequencing (GBS) protocol developed for maize (Elshire et al., 2011) using a 5‐bp single‐cutter restriction enzyme, *Ape*KI. Single‐end sequencing generated 100‐bp reads on a single lane of an Illumina HiSeq 3000 flow cell at the Center for Genome Research and Biocomputing at Oregon State University. Base calling was performed in Casava v1.8 (Illumina, San Diego, CA).

### 2.3 Dataset construction and bioinformatics

To increase genome coverage and read depth for SNP discovery, we complemented our new Illumina GBS‐generated dataset above with a published dataset from Schilling et al. (2014) including *P. tremuloides* and the outgroup *P. trichocarpa* Torr. & A. Gray ex Hook. Combining datasets was justified as Schilling et al. (2014) employed the same *Ape*KI GBS protocol herein, with samples prepped in two multiplexed libraries each containing 96 individuals and sequenced on separate Illumina HiSeq 2000 lanes. Schilling et al.’s (2014) dataset comprised 104 *Populus* samples from individuals representing six additional U.S. and Canadian *P. tremuloides* subpopulations (Figure 1) and 1 *P. trichocarpa* subpopulation (additional details in Appendix S1 and Data S1 of the Supporting Information), plus technical replicates from a subset of 45 individuals.

We conducted reference‐based assembly, SNP discovery, and genotyping using the TASSEL‐GBSv2 pipeline (Glaubitz et al., 2014) in TASSEL v5.0 (Bradbury et al., 2007). Unfiltered Illumina data were demultiplexed by barcode, trimmed to 64 bp, and stored in bit format to reduce computational time. Identical reads were collated as haplotypes or “tags” (Lu et al., 2013). To allow singletons for improving demography/gene flow inferences, we set the minimum *k*‐mer count across taxa and read counts per tag to 1. For the reference genome, we used the updated annotated genome for *P. tremuloides* (v1.1; “Potrs01b,” ~480 Mbp of sequence) available from The *Populus* Genome Integrative Explorer website (http://www.popgenie.org/; Sjödin et al., 2009; Sundell et al., 2015). Reads were mapped to the reference genome based on sequence similarity using the Burrows–Wheeler alignment tool, bwa v0.7.17 (Li & Durbin, 2009). SNPs were called from tag alignments starting at the same physical position along the reference genome and filtered using default 10% minimum locus coverage (proportion of individuals) and minimum 0.01 minor allele frequency (MAF) settings. To generate a “final” SNP set for downstream analyses, we removed indels and four problematic individuals with low barcode assignment success, and we used vcftools v0.1.14 (Danecek et al., 2011) to filter out SNPs not meeting the following criteria: (a) biallelic loci, (b) minimally two individuals per allele, (c) MAF ≥ 0.0025, and (d) minimum locus coverage >50%. We also evaluated potential effects of including technical replicates, as described in Appendix S1.

### 2.4 Population genetic diversity, structure, and admixture

We used two methods to infer overall patterns of population genetic structure and individual ancestry. First, we used the model‐based approach implemented in ADMIXTURE v1.3.0 to infer individual ancestries in a maximum‐likelihood (ML) framework that outperforms STRUCTURE (Pritchard, Stephens, & Donnelly, 2000) in computational efficiency for large, genome‐wide SNP datasets such as ours (Alexander, Novembre, & Lange, 2009; Alexander & Lange, 2011). We estimated ancestry coefficients (*Q*) for each individual in 10 replicate ADMIXTURE runs for each of *K* = 1–10 current/ancestral gene pools or “clusters.” We also performed two replicate runs of 50‐fold cross‐validation for *K* = 1–10 to determine potential error in ancestry estimation of each *K*. We then plotted the errors and identified the *K*‐value having the lowest cross‐validation error as the best *K*. Each individual was assigned to the cluster for which it had the highest corresponding *Q*‐value in the ancestry coefficient matrix for the best‐*K* model. Second, we estimated genetic clusters and membership probabilities using model‐free discriminant analysis of principal components (DAPC; Jombart, Devillard, & Balloux, 2010) in the adegenet package (Jombart & Ahmed, 2011) of R v3.5.0 (R Core Team, 2018). Unlike ADMIXTURE, DAPC does not assume panmixia within genetic clusters or linkage equilibrium among loci. We objectively identified the appropriate number of PCs using the “xvalDapc” cross‐validation procedure, which evaluates prediction success of using DAPC on a training set of 90% of observations from each local subpopulation to group the remaining 10% of observations (Jombart et al., 2010). Results were compared for the number of PCs minimizing mean‐squared error, and the maximum number of PCs with >90% prediction success.

We evaluated general patterns of genetic diversity by calculating observed (*H*_O_) versus expected (*H*_E_) heterozygosity, gene diversity (*H*_S_), *F*_IS_, and counts of heterozygote and singleton alleles on per‐individual and per‐locus bases, and compared these among local subpopulations and ADMIXTURE clusters. Calculations were conducted using vcftools (Danecek et al., 2011), adegenet (Jombart & Ahmed, 2011), and hierfstat (Goudet, 2005). Given *P. tremuloides* likely experienced a dramatic northward range expansion following the LGM (e.g., Callahan et al., 2013; Jackson et al., 2000), we tested for prevailing latitudinal or longitudinal clines in *H*_O_ and *H*_S_ using generalized linear models (GLMs) estimated in R for the species, and within each genetic cluster herein (see Results). We used these two heterozygosity measures because they are common metrics of genetic variation and they are complementary with respect to one another: Whereas *H*_O_ reflects observed heterozygosity*, H*_S_ is a biased estimator that accounts for the fact that the proportion of closely related (e.g., inbred) individuals increases with increasing numbers of sampled alleles (Nei, 1987). We expected negative linear relationships in populations that experienced extensive postglacial expansion consistent with the trailing‐edge hypothesis (Excoffier et al., 2009; Hampe & Petit, 2005; Hewitt, 1996). Approximate GLM R^2^ values were calculated using the “nagelkerke” function of the rcompanion package (Mangiafico, 2019). We calculated pairwise Nei's (1972) *D* and *F*_ST_ (unbiased estimator of Weir & Cockerham, 1984) genetic distances between clusters and subpopulations in StAMPP (Pembleton, Cogan, & Forster, 2013). We assessed the differentiation of clusters further using a hierarchical analysis of genetic variance in hierfstat (“varcomp.glob” and “boot.vc” functions), with trees (“ind”) nested within local subpopulations (“pop”), nested within genetic clusters (“clust”), and significance of *F‐*statistics was estimated using 95% confidence intervals (CIs) from 100 bootstrap pseudoreplicates.

To evaluate spatial patterns of genetic variation and test the prediction of the stable‐edge hypothesis that southwestern *P. tremuloides* populations exhibit isolation by distance (IBD) due to long‐term migration–drift equilibrium (Callahan et al., 2013; Hampe & Petit, 2005), we tested for IBD using Mantel randomization tests and GLMs of linearized *F*_ST_ [=*F*_ST_/(1 − *F*_ST_)] versus log‐geographical distance (after Rousset, 1997). Tests used straight‐line geographical distance between sampling locations estimated while accounting for the curvature of the earth's surface (Vincenty inverse solution) in Imap (Wallace, 2012). Mantel significance (*α* = 0.05) was assessed using 10,000 randomizations of the data in ade4 (Dray & Dufour, 2007). IBD tests were conducted globally and within each ADMIXTURE cluster.

### 2.5 Ploidy and possible effects on genetic inferences

Analyses of microsatellite DNA, GBS SNPs, and flow cytometry data have shown that *P. tremuloides* populations vary in composition from diploid to tetraploid or aneuploid (Gompert & Mock, 2017; Mock et al., 2012). While most individuals or clones are diploid, the within‐population proportion of triploids is higher (up to 69%) in southwestern portions of the species geographical range, including western Pacific‐coastal mountains and the Rocky Mountains (Mock et al., 2012). To assess potential impacts of polyploidy on our inferences, we first inferred ploidy levels in our dataset and then asked whether polyploids have substantially influenced *P. tremuloides* inference of genetic structure or genetic diversity metrics; hence, the above tests depend on such metrics.

We used nQuire v1 (Weiß, Pais, Cano, Kamoun, & Burbano, 2018) to directly infer ploidy levels from Illumina reads mapped to the reference genome. nQuire models the distributions of base frequencies at biallelic SNPs using a Gaussian mixture model (GMM) and estimates parameters of the model under ML for an unconstrained “free” model versus three models each constraining the underlying Gaussians to expectations for diploidy, triploidy, and tetraploidy. Subsequently, model selection on maximized log‐likelihoods is used to determine the optimal model for the data. First, to generate BAM files with mapped Illumina reads (not output by TASSEL above), we trimmed the raw reads for each sample and mapped them to the *P. tremuloides* reference genome using bwa, as automated in dDocent v1 with default parameters (Puritz, Hollenbeck, & Gold, 2014). Second, for all samples meeting the default criteria of multiple biallelic alleles at minimum 0.2 frequency and 10 × coverage, we ran nQuire by “denoising” the BAM reads, and then running the models and conducting model selection using the “lrdmodel” function. We summarized and graphically plotted per‐individual read frequency histograms and model selection results in R. We used the R maps package v3.3.0 (Becker, Wilks, Brownrigg, Minka, & Deckmyn, 2018) to plot pie charts of the frequency of ploidy levels within *P. tremuloides* populations over our sampling sites.

If most or all individuals in a population are polyploids, then genetic diversity, including *H*_O_ and *H*_S_ estimates, should be greater relative to expectations for an equally sized diploid population, due to increased chromosome number and more limited effects of genetic drift (Meirmans, Liu, & Tienderen, 2018). Thus, our GLM cline and IBD analyses above could potentially have been compromised by significant positive relationships between the incidence of polyploidy and our diversity metrics within populations. We tested this hypothesis by testing for significant positive relationships between the number of polyploids (triploids plus tetraploids) and *H*_O_ within each local *P. tremuloides* population, using GLMs implementing negative binomial and Poisson regressions in R (based on goodness‐of‐fit chi‐square tests with 1 degree of freedom; details in Appendix S1). Approximate *R* ^2^ values were calculated under ML in rcompanion. We also tested whether the presence of polyploids unduly influenced our GLM tests for clinal patterns of *H*_O_ and *H*_S_ with latitude and longitude by rerunning those analyses on a dataset excluding individuals scored as polyploids by nQuire. The presence of polyploids in our sample could also have influenced our ADMIXTURE analyses, for example, if one genetic cluster was entirely polyploid, or if nonintrogressed, paralogous site patterns from multiple chromosomes were mistaken as admixture. We evaluated these possibilities by rerunning ADMIXTURE on a dataset excluding individuals scored as polyploids.

### 2.6 Tree graph analyses with admixture

Before analyses, we removed three individuals with ≳50% missing data (CSS11, GCB5, and SFRG5‐4‐1; Data S1), yielding 180 samples. We used TreeMix v1.13 (Pickrell & Pritchard, 2012) to infer a ML tree topology of relationships among genetic clusters identified during our ADMIXTURE and DAPC analyses (see Results) while accounting for admixture among ancestral *Populus* populations. In the first set of analyses, the *P. trichocarpa* sample was used to fix the root, we accounted for linkage disequilibrium by using 500‐bp blocks of SNPs, and a no‐migration run was followed by a series of replicated runs sequentially adding migration events (–m flag). Migration edges were added until the proportion of SNP variance explained by the model reached ≥99.8% (Pickrell & Pritchard, 2012). We evaluated the consistency and significance of migration edges by running five replicate runs at the final migration level while estimating standard errors of migration weights. We estimated nodal support using 500 bootstrap pseudoreplicates with *k* = 500 bp blocks of contiguous SNPs, and we evaluated whether supported migration edges significantly improved the fit to the data using the default jackknifing procedure (Pickrell & Pritchard, 2012). Support for migration edges or novel admixture patterns was also assessed using plots of the residual fit of the tree graph. We evaluated potential effects on our TreeMix results of the substantial proportion of missing data in the outgroup sample (~48%) by rerunning the TreeMix procedure above while excluding the outgroup sample, producing unrooted tree topologies. We also conducted a performance analysis assessing the impacts of *k* (block size) on our results, by reanalyzing the full dataset over the following range of *k* values: 10, 100, 250, 500, 750, 1,000, 2,000, and 5,000 bp.

### 2.7 Ecological niche modeling

We used *P. tremuloides* occurrence data from throughout the species native distribution from Worrall et al. (2013), as well as from our genetic sampling sites. As the dataset contained >100,000 occurrence records and such a large dataset would likely carry elevated geographical or environmental space biases (Boria, Olson, Goodman, & Anderson, 2014; Reddy & Dávalos, 2003), we decreased the number of records by spatially filtering occurrences located ≤10 km from other occurrences using the spThin R package (Aiello‐Lammens, Boria, Radosavljevic, Vilela, & Anderson, 2015). The filtered dataset comprised 14,146 occurrences and was used in subsequent analyses. Our ENM analyses employed environmental data layers for 19 bioclimatic variables at a resolution of 2.5 arc‐minutes, available in the WorldClim 1 dataset (Hijmans, Cameron, Parra, Jones, & Jarvis, 2005). The calibration area for the models was created as a 1.5° buffer around the minimum convex polygon encompassing all occurrence sites in the filtered dataset (sensu Anderson & Raza, 2010; Barve et al., 2011).

To calibrate our models, we employed the maximum entropy method implemented in MaxEnt ver. 3.3.3k76 (Phillips, Anderson, Dudík, Schapire, & Blair, 2017; Phillips, Anderson, & Schapire, 2006; Phillips & Dudík, 2008). Given the importance of evaluating model performance with spatially independent data and balancing model complexity and predictive power (Radosavljevic & Anderson, 2014; Warren & Seifert, 2011), we fine‐tuned MaxEnt using ENMeval (Muscarella et al., 2014) accessed through the ENMwizard R package (Heming, Dambros, & Gutiérrez, 2018). We evaluated models using a geographical partition scheme, and we optimized two important MaxEnt parameters that impact model complexity and predictive power: the regularization multiplier (RM) and feature classes (FCs) (Muscarella et al., 2014). To optimize RM and FCs, we calibrated preliminary models employing a range of settings under the geographical “block” partitioning scheme (Muscarella et al., 2014), but only varying the RM and FC values. We used 8 values of RM from 0.5 to 4.0, incremented by 0.5. For each RM, we conducted 15 preliminary analyses, one with each of the following feature classes or combination thereof: L, P, Q, H, LP, LQ, LH, PQ, PH, QH, LPQ, LPH, LQH, PQH, and LPQH, where “L” is linear, “P” is product, “Q” is quadratic, and “H” stands for hinge. In total, 120 preliminary models were built to select the best settings, including RM and FC combinations. Model selection was based on the corrected Akaike information criterion (AICc). Model omission rates calculated with the 10th percentile and minimum training presence thresholds, and the area under the receiver operating characteristic curve (AUC), were also used as secondary criteria for model selection (Peterson et al., 2011; Warren & Seifert, 2011). Selected MaxEnt parameters (see Data S2) were used to calibrate a final model using occurrences in the final filtered dataset, but without a geographical partition scheme. The final model was projected onto multiple climatic and paleoclimatic scenarios from late Pleistocene to present (Table 1) within a geographical area covering an extent of 12.5°–75.9° N and 47.0°–172.2° W.

**Table 1:** List of climatic scenarios onto which *P. tremuloides* ecological niche models were projected. Raster files were obtained from WorldClim (Hijmans et al., 2005). Time period is given either as date in the Gregorian calendar system or as thousands of years ago (ka) in the case of paleoclimatic environmental reconstructions

Climatic scenario	Time period	GCM^a^	Resolution^b^	References
Present	1960–1990	–	2.5 arc‐minutes	Hijmans et al. (2005)
Mid‐Holocene	~6 ka	CCSM4	2.5 arc‐minutes	Gent et al. (2011)
		IPSL‐CM5A‐LR		Kageyama et al. (2013a,2013b)
		MIROC‐ESM		K‐1 model developers (2004)
		MPI‐ESM‐P		Stevens et al. (2013)
Last Maximum Glacial	~22 ka	CCSM4	2.5 arc‐minutes	Gent et al. (2011)
		MIROC‐ESM		K‐1 model developers (2004)
		MPI‐ESM‐P		Stevens et al. (2013)
Last Interglacial	~120–140 ka	NCAR‐CCSM	0.5 arc‐minutes	Otto‐Bliesner, Marshall, Overpeck, Miller, and Hu (2006)

^a^ General circulation model names.

^b^ Resolutions of corresponding raster files.

We conducted a second set of ENM analyses to infer potential past to present geographical distributions of genetic clusters within *P. tremuloides* (see Results), and to test for different range shifts predicted under the stable‐ versus trailing‐edge hypotheses. Calibration areas should not include regions that species/lineages cannot disperse to due to geographical or ecological barriers (Anderson & Raza, 2010). Assuming intraspecific genetic clusters have similar dispersal capabilities, we defined calibration areas for each cluster by building minimum convex polygons using the coordinates of genetic sampling sites exclusive to each cluster. We also excluded areas pertaining to other clusters (e.g., possible focal cluster absence due to competitive exclusion) and areas with species occurrences pertaining to unidentified lineages, that is, areas not covered by our genetic sampling. Procedures were performed in R using ENMwizard and raster (Hijmans, 2017; see details in Appendix S1).

## 3 RESULTS

### 3.1 Dataset construction, SNP discovery, and SNP filtering

Illumina sequencing on our *Ape*KI GBS library yielded a total of 382 million reads after initial base calling, with an average of 3.9 million reads per sample. The total number of “good” barcoded reads with clear sample assignments was 321 million (85%), and the total number of unique tags retained was 17.5 million (5.5%). Out of 96 samples, 17 samples failed quality checks, based on having <10% of mean reads per sample; thus, data from 79 samples were retained. Combining these data with 313 million raw reads from Schilling et al. (2014) yielded a total of 634 million barcoded reads, with an average of 3.1 million reads per sample. Reference‐based assembly and SNP calling in the TASSEL‐GBSv2 pipeline yielded 56,246 SNPs, and results were 99.4% similar to the initial SNPs when technical replicates were excluded (Fig. S2 of Appendix S1). After filtering in vcftools and quality controls, the final variant set contained 34,796 SNP loci for 182 *P. tremuloides* individuals from 36 sites and one *P. trichocarpa* individual. Per‐individual read depth of coverage averaged 13.95 × per locus and overall, and the final data matrix was highly complete with on average ~15% missing data per individual (Figs. S3 and S4). The final dataset showed considerable genetic variation, with global *F*_ST_ and *F*_IS_ over all loci by subpopulation (Weir & Cockerham, 1984) of 0.148 and 0.186, respectively. Most genetic variation was present in the ingroup, and removing the *P. trichocarpa* outgroup sample yielded nearly identical global *F*‐statistics. Genetic diversity at these loci was moderate, for example, with mean observed heterozygosity (*H*_O_) of 0.13 and mean overall gene diversity (*H*_t_) of 0.18 (details in Table S1).

### 3.2 Population genetic diversity, structure, and admixture

**Figure 2:**
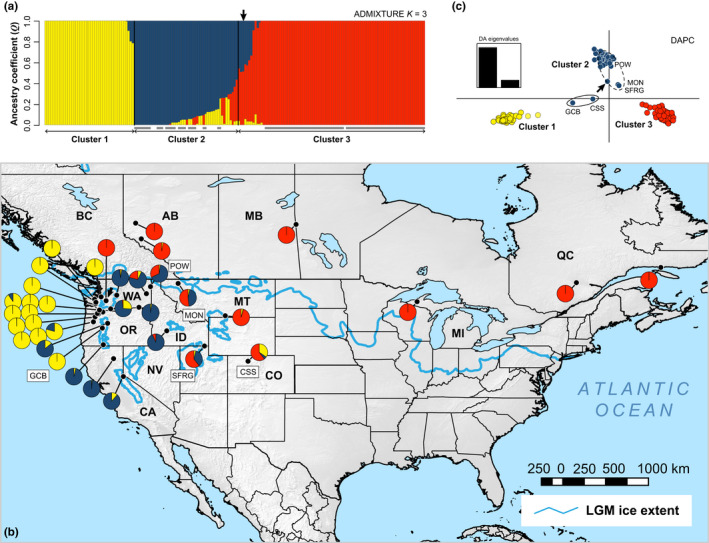
Population genetic structure of *P. tremuloides* and outgroup (*P. trichocarpa*) individuals inferred from 34,796 SNP loci. Results are shown for ADMIXTURE (Alexander et al., 2009) assignment of individuals to each of *K* = 3 clusters (a) plotted onto topographic map of the study area (b) and a layer indicating the extent of glacial ice sheets during the LGM. Pie charts show the per‐site average ancestry assignment to each genetic cluster (*Q* ≥ 0.5), and colors correspond to the ADMIXTURE barplot. Discriminant analysis of principal components (DAPC; Jombart & Ahmed, 2011; Jombart et al., 2010) yielded a classification that was 95% similar to ADMIXTURE, with results plotted along the first and second discriminant functions and colored by cluster (c). Intermediate, encircled individuals in panel C were assigned to cluster 1 (solid ellipse) or cluster 3 (dotted ellipse) in ADMIXTURE. Positions of the outgroup sample are indicated by bold arrows throughout

Our ADMIXTURE and DAPC clustering analyses each identified three genetic clusters (Figure 2). Cross‐validation error estimates for the ADMIXTURE models decreased rapidly from *K* = 1 to reach a low point at *K = *3, the “best” *K*, and then increased steadily to higher levels (Fig. S5 of Appendix S1). In the final ADMIXTURE model (Figure 2a), cluster 1 was located along the Pacific Northwestern coast and Cascades Range in Washington and Oregon. Consistent with predictions of the inland dispersal hypothesis, H_4_, cluster 2 had a disjunct distribution between the eastern Cascades–Sierra Nevada mountain ranges and the Northern Rocky Mountains, but exhibited limited genetic divergence (Figure 2b, c). Cluster 3 was present across the remainder of the study area, including the U.S. Rocky Mountains (Northern Rockies to Southern Rockies) but mostly across southern Canada (Figure 2b). Individuals in clusters 2 and 3, especially *P. tremuloides* from sites CSS, GCB, SFRG, POW, and MON (Figure 2b, c), had admixture proportions consistent with putative genomic backgrounds involving introgression (*Q* = 0.15–0.5 for at least one cluster). The *P. trichocarpa* outgroup sample had admixture proportions equally divided between clusters 2 (*Q* = 0.48) and 3 (*Q* = 0.52), which could reflect noise in the data, given these taxa have been isolated for a long period of time. Geographical gradients in *Q‐*values indicated admixture between neighboring clusters and little or no admixture between disjunct clusters 1 and 3. The only exception was the CSS population in east‐central Colorado, which had individuals partially assigned to all three clusters. Population structure was not driven by merging datasets, as individuals from the Schilling et al. (2014) dataset did not form their own cluster, but were interspersed across clusters 2 and 3.

The *k*‐means clustering step of DAPC identified three clusters as the best solution based on the BIC inflection point (Fig. S6). During DAPC cross‐validation, prediction success peaked at 20 retained PCs and remained above 90% up to 100 PCs, indicating that lower values optimally minimized error, but retaining 100 PCs minimized error while maximizing information content (Fig. S7). DAPC results were identical across 20–100 PCs and driven by the same SNPs in loading plots of each allele (Figs. S7 and S8); thus, only results based on 100 PCs are presented. The final DAPC used two discriminant functions and yielded three clusters that were clearly differentiated along the first axis, matched the geographical pattern of the ADMIXTURE clusters (Figure 2c), and were 95% similar to those from ADMIXTURE (additional details in Appendix S1). Thus, we took the ADMIXTURE results as our best estimate of distinct genetic clusters and used them as a priori groups in subsequent genetic analyses.

**Figure 3:**
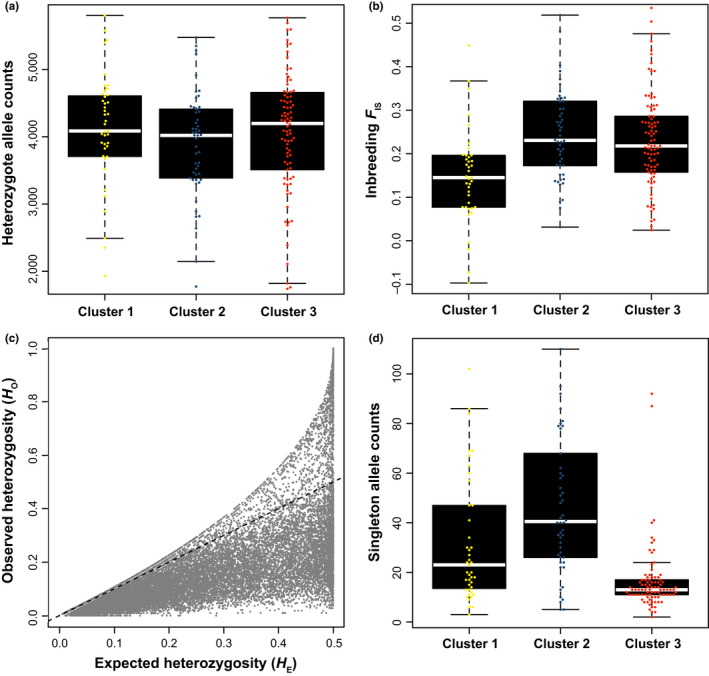
Genetic patterns of heterozygote and singleton alleles within and among *P. tremuloides* genetic clusters. Panels present ranges of observed numbers of heterozygote genotypes (a); ranges of inbreeding *F*_IS_, a measure of heterozygote deviation from Hardy–Weinberg expectations (b); observed heterozygosity (*H*_O_) plotted against expected heterozygosity (*H*_E_), with deviations mainly below the dotted identity (1:1) line (c); and ranges of observed numbers of singleton genotypes

Genetic diversity patterns were consistent with deviations from Hardy–Weinberg equilibrium due to pronounced population structure, with heterozygosity being substantial and similar among ADMIXTURE clusters (Figure 3a) but lower than expected under random mating (Figure 3c). Cluster 1 exhibited substantial heterozygosity, relatively fewer singletons, and lower inbreeding *F*_IS_ consistent with limited admixture (Figure 3b). By contrast, clusters 2 and 3 had relatively elevated inbreeding *F*_IS_, possibly related to their higher levels of admixture with other clusters. Cluster 3 also exhibited the lowest per‐individual counts of singleton SNPs or private genetic variation (Figure 3). Reanalyses excluding putatively admixed cluster 2 and 3 edge populations (*Q*_max_ < 0.75) yielded nearly identical results (Fig. S9), indicating that hybridization likely has not biased our genetic diversity estimates.

Pairwise Nei's *D* estimates, pairwise *F*_ST_ estimates (Table S1), and heatmaps plotted against clustering trees of the pairwise distances (Figs. S10 and S11) suggested that cluster 3 was the most divergent from other clusters. Consistent with this and with phylogenetic results below, *F*_ST_ was also lowest between clusters 1 and 2. Hierarchical genetic differentiation was moderate and significant between clusters relative to the whole species (*F*_clust/total_ = 0.092, 95% CIs: 0.090–0.094) and between subpopulations within clusters (*F*_pop/clust_ = 0.089, 95% CIs: 0.088–0.091; Table S2), and these two levels made relatively equal contributions to genetic variance partitioning among subpopulations (*F*_pop/total_ = 0.173, 95% CIs: 0.170–0.176).

**Figure 4:**
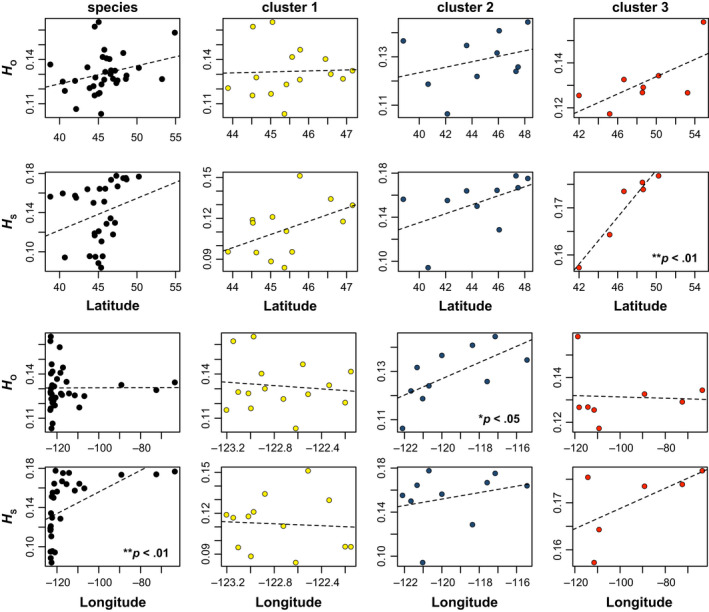
Latitudinal and longitudinal clines in SNP genetic diversity within *P. tremuloides* subpopulations. Linear relationships of observed heterozygosity (*H*_O_) and gene diversity (*H*_S_) with latitude (top two rows) and longitude (bottom two rows) are presented for *P. tremuloides* as a whole, and for each ADMIXTURE‐inferred genetic cluster. Each dot represents a population and is colored to match the colors of genetic clusters defined in Figure 2, and regression lines are drawn from fitted values of generalized linear models. For significant relationships, *p*‐values are given in the lower right of the plot

In line with predictions of the stable‐edge hypothesis, *H*_O_ and *H*_S_ relationships with latitude and longitude were mostly nonsignificant, indicating a lack of clear genetic signals of directional spatial expansions in clusters 1 and 2 in west‐central portions of the species range (Figure 4). None of the GLMs provided support for the trailing‐edge hypothesis: Instead of negative genetic diversity clines, genetic diversity metrics exhibited either flat or positive trends over latitude and longitude. The only significant GLMs revealed positive scaling of genetic diversity with increasing latitude within cluster 3 (*H*_S_: Cox and Snell *R* ^2^ = 0.92, *t* = 6.92, *df* = 5, *p* = .0023), and positive scaling of genetic diversity with increasing longitude at the species level (*H*_S_: Cox and Snell *R* ^2^ = 0.27, *t* = 3.22, *df* = 29, *p* = .0033) and within cluster 2 (*H*_O_: Cox and Snell *R* ^2^ = 0.44, *t* = 2.51, *df* = 9, *p* = .036).

Contrasting results of Callahan et al. (2013), who reported significant IBD in their southwestern *P. tremuloides* cluster, we found no evidence for significant IBD overall, or within any of the ADMIXTURE clusters. Although linearized genetic distance declined with log‐geographical distance in km between sites when analyzing the full dataset (Mantel's *r* = −0.25), the pattern was diffuse (*R* ^2^ = 0.063, *p* = .99). Relationships were even more diffuse but slightly positive during separate analyses of clusters 1–3 (*p* > .05, Mantel's *r* range = 0.07–0.23; Fig. S12 and Table 2).

**Table 2:** Results of Mantel tests for isolation by distance investigating relationships between *F*_ST_ and geographical distances within *P. tremuloides*

Group	Mantel test	*r*	*R* ^2^	*p*‐value
*P. tremuloides*	linearized *F*_ST_ × log[straight‐line dist. (km)]	−0.252	0.063	.99
Cluster 1	linearized *F*_ST_ × log[straight‐line dist. (km)]	0.228	0.052	.13
Cluster 2	linearized *F*_ST_ × log[straight‐line dist. (km)]	0.103	0.011	.27
Cluster 3	linearized *F*_ST_ × log[straight‐line dist. (km)]	0.071	0.005	.33

Note

Results are shown for simple Mantel (1967) tests performed for the species as a whole, and for each of the ADMIXTURE‐inferred genetic clusters within *P. tremuloides* presented in Figure 2. Mantel's *r* is the standardized test statistic and is equivalent to Pearson's *r*, and *R* ^2^ is the coefficient of determination.

### 3.3 Polyploid inference and negligible effects of polyploidy on genetic inferences

We successfully distinguished ploidy levels from mapped reads for 161 samples (88%) meeting the read criteria for biallelic SNPs using the GMM approach in nQuire. The overwhelming majority of these samples were diploid (*n* = 139; 86.3%), while only 20 individuals (12.4%) from 10 local populations were triploid and only 2 individuals (1.2%) from 2 populations were tetraploid (additional details in Appendix S1, Fig. S13, and Data S1). Consistent with previous studies (e.g., Mock et al., 2012), a substantial proportion of triploids (45%) came from populations of the Cascades and the Rocky Mountains; remaining triploid and tetraploid individuals were from northern populations across Canada (Figure 1). Analyses were generally insensitive to sample sizes, as polyploid individuals were discovered in populations with 2 to 20 samples; however, the maximum triploid proportion of 50% in one local population, SFRG, was a sampling artifact, as it had only 2 individuals. Against expectations, and consistent with limited effects on our downstream genetic inferences and hypotheses tests, a negative binomial GLM evaluating whether polyploid counts within populations could be predicted by *H*_O_ was negatively sloping, diffuse, and nonsignificant (Cox and Snell *R* ^2^ = 0.0023, *z* = −0.33, *df* = 33, *p* = .79; additional details in Fig. S14 and Appendix S1). Also consistent with limited effects of polyploids on our inferences, results from GLM tests for latitudinal and longitudinal clines in genetic diversity based on putative diploids were highly similar to results for the full dataset, with only one of 16 models exhibiting a clear change in slope (Fig. S15). After removing putative polyploid individuals, reanalyzing our dataset in ADMIXTURE yielded results (Fig. S16) that were nearly identical to the original ADMIXTURE results for the full dataset, suggesting that the presence of polyploids did not majorly influence our genetic structure or admixture inferences.

### 3.4 Tree graph analyses with admixture

**Figure 5:**
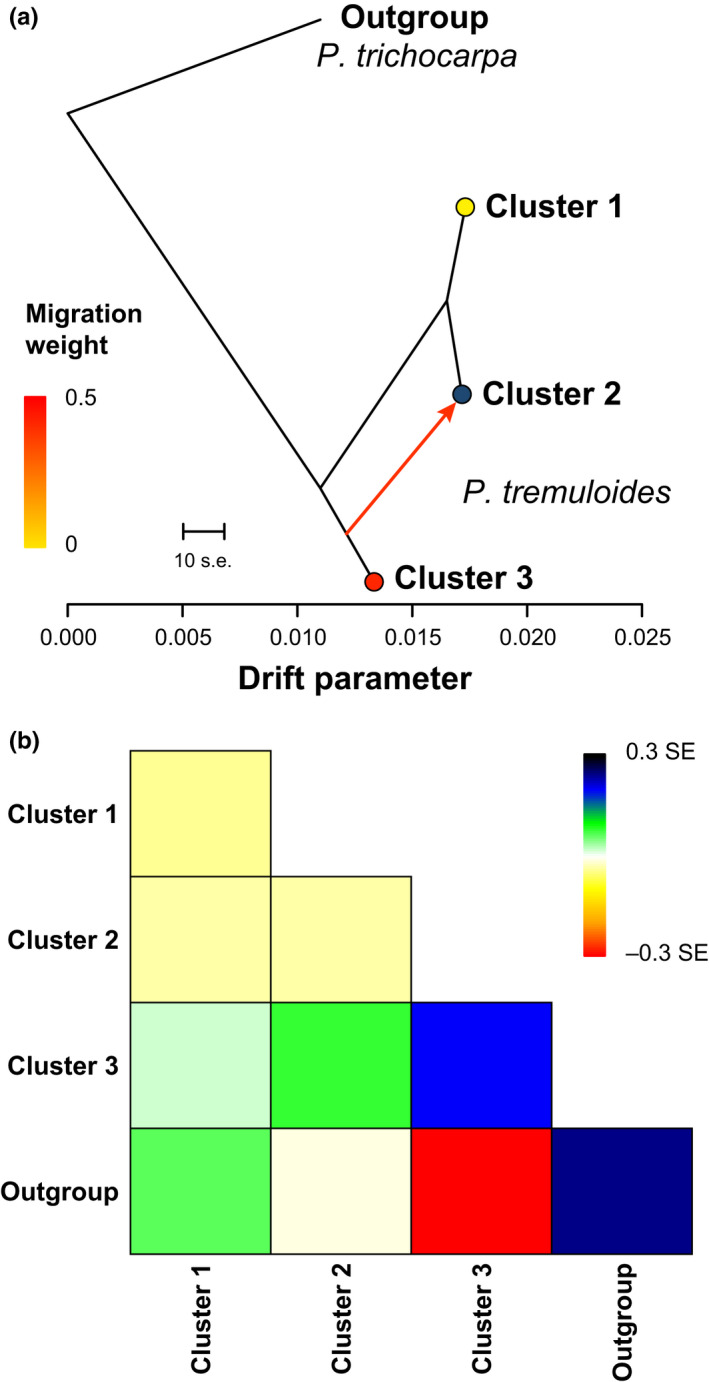
Tree graph relationships among *P. tremuloides* genetic clusters, and admixture from cluster 3 into cluster 2. The final ML tree graph inferred in TreeMix v1.13 (Pickrell & Pritchard, 2012) allowing a single migration event (a) is presented with the migration event arrow colored by migration weight and clusters indicated by colors corresponding to Figure 2. Scale bar: 10 times the mean standard error in the sample covariance matrix. To show putative patterns of admixture not apparent from the tree and migration edges, the residual fit of the graph is plotted and colored by the standard error color palette (b)

The proportion of SNP variance explained by the tree graph estimated without migration in TreeMix was 99.7% and surpassed the target of 99.8% when one migration edge was added (“m1” model; proportion of variance explained = 100%). Subsequently, five independent runs using the final m1 model consistently yielded the same tree topology and significant migration edge from ADMIXTURE cluster 3 into cluster 2 (*p* < .001). The final m1 run that maximized the log‐likelihood of the model yielded a tree placing clusters 1 and 2 more closely related to one another than to cluster 3, and that was strongly supported by bootstrap proportions (Figure 5a). The corresponding residual plot (Figure 5b) agreed with patterns of admixture inferred from ADMIXTURE and DAPC results but suggested moderate admixture between cluster 1 and *P. trichocarpa*, despite current crossability barriers between these species. The analysis excluding the outgroup sample yielded a similar migration event from the ancestral cluster 3 population into cluster 2, with slightly different residuals (Fig. S17), suggesting that inclusion of the outgroup had no adverse effects. Likewise, TreeMix performance was similar over *k* values varying by two orders of magnitude for the analysis of the full dataset, indicating that results were insensitive to our choice of block size parameter to account for LD (Fig. S18).

### 3.5 Ecological Niche Modeling

ENMeval analyses identified RM = 0.5 and a combination of product and hinge feature classes as the best‐performing parameters for calibrating the final ENM. These parameters yielded a single “best” candidate model with the lowest AICc score (135,032.19) and an AICc weight of ~1.0. This best‐supported model had mean omission rates of 0.24 and 0.06 for the 10th percentile and the lowest presence training thresholds, respectively, as well as a mean test AUC of 0.733. Values of these diagnostic metrics are provided for all candidate models, in Data S2.

**Figure 6:**
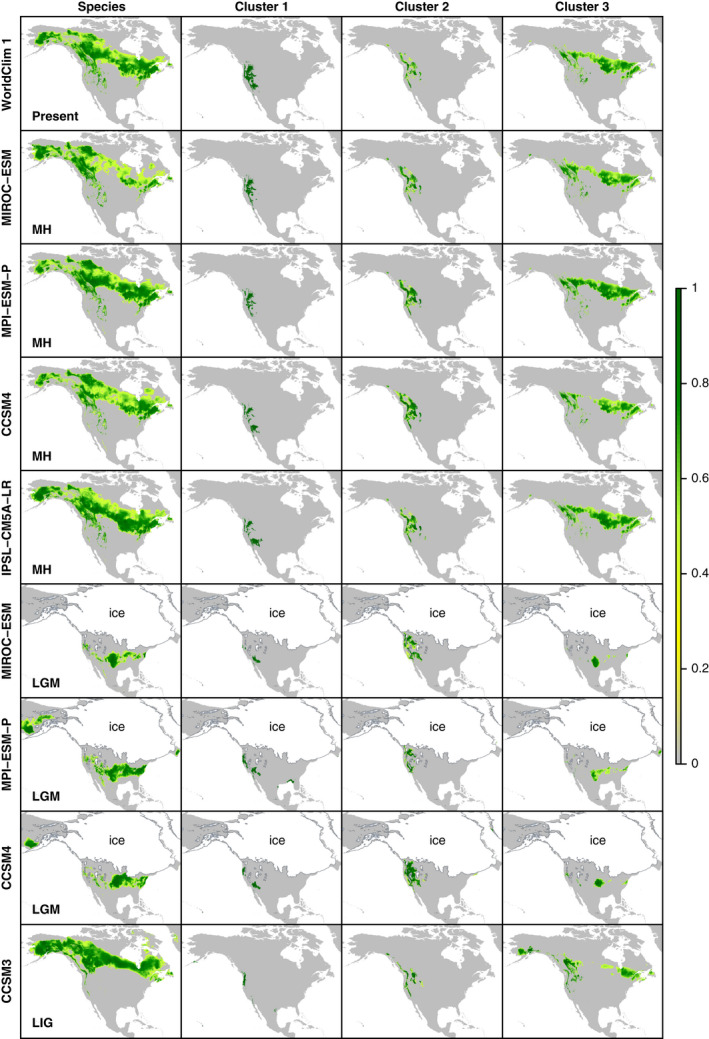
Predicted geographical distributions of *P. tremuloides* and its intraspecific genetic clusters from late Pleistocene to present. MaxEnt models predicting present‐day ranges of *P. tremuloides* and clusters 1–3 (top row) based on WorldClim v1 (Hijmans et al., 2005) were projected onto data layers for three late Pleistocene time slices described in Table 1: Mid‐Holocene (MH), Last Glacial Maximum (LGM), and Last Interglacial (LIG). Predictions are shown as color gradients from low (gray–dark yellow) to high (dark green) habitat suitability based on logistic output of the models (probability from 0 to 1), and the extent of land during each time period is shown in gray. The extent of ice sheets during the LGM is indicated in white

Analyses based on objectively defined calibration areas (Fig. S19 of Appendix S1) and projecting the final model onto geographical space revealed large areas with suitable conditions for *P. tremuloides* during the present, ~6 ka in the Mid‐Holocene (MH), and ~ 125 ka during the LIG, while suitable climatic conditions for the species were restricted to a much smaller area ~22 ka during the LGM (Figure 6 and Figure S20). During present‐day, MH, and LIG periods, areas with suitable conditions for *P. tremuloides* showed a similar pattern, nearly all being located in northern North America and mainly covering modern‐day Alaska, much of Canada, and small patches throughout the northwestern and western United States (Figure 6 and Figure S20a, b, and d). By contrast, predicted suitable conditions during the LGM only occurred in the modern‐day United States—mostly in the central and eastern United States, but also in small scattered disjunct areas mainly throughout the southern Cascade Range, northern Sierra Nevada, Great Basin, and mid‐ to Southern Rockies (Figure 6 and Figure S20c). These general patterns were consistent among results based on different general circulation models (Table 1) and indicated an overall pattern of southward range contraction to one or more refugia in south‐central areas of North America (Figure 6).

The minimum convex polygon approach to defining areas for calibrating models of *P. tremuloides* clusters yielded areas from which *n* = 11, 45, and 1504 filtered occurrence points were extracted for clusters 1, 2, and 3, respectively (Fig. S19). Projecting final models for each cluster onto geographical space indicated that the overall pattern of range dynamics within *P. tremuloides* resulted from distinct late Pleistocene range shifts of the clusters relative to one another and to the species as a whole. Broadly consistent with the stable‐edge hypothesis, cluster 1 predictions exhibited relative stability over the last glacial cycle, with contiguous or disjunct areas of high‐predicted habitat suitability signaling refugial areas along the Pacific Northwestern coast, Olympic Peninsula, and Aleutian Islands (Figure 6). By contrast, predicted suitable habitat areas for cluster 2 extended along coastal mountain ranges and the Northern Rockies for much of the late Pleistocene, but expanded to cover much of northern North America during the LGM and progressively contracted to its modern geographical range through an intermediate MH stage. Predicted interglacial (LIG and present) suitable areas for cluster 3 were scattered across the Rockies and parts of the Great Basin to the west, with a more or less contiguous area across southern Canadian boreal forests from Saskatchewan east to the Atlantic coast (Figure 6). However, closely matching trailing‐edge hypothesis predictions, LGM suitable areas for cluster 3 fully shifted to mid‐latitudes in the continental interior (similar to species‐level results), with two minor‐potential refugial areas located to the west and east. Consistent with gene flow between clusters 2 and 3 in our TreeMix results, paleodistribution modeling indicated overlapping areas of suitable habitat for these lineages during late Pleistocene interglacial periods, including LIG, MH, and present‐day areas in the Northern Rockies, Middle Rockies, and scattered areas of the intermountain west (Figure 6). Suitable areas for the genetic clusters were again consistent across MaxEnt analyses, suggesting they were robust to differences among circulation models.

## 4 DISCUSSION

We inferred the phylogeographic history of *P. tremuloides* by combining broadscale inferences of population structure, admixture, and ploidy based on genome‐wide SNP data (Elshire et al., 2011) with spatially explicit predictions of the past to present geographical distributions of the species and its intraspecific lineages using ENM hindcasting (Peterson et al., 2011; Waltari et al., 2007). Under this framework, we found strong evidence for significant patterns of population divergence and admixture among three intraspecific genetic clusters, including a new and genetically distinct lineage of Pacific‐coastal aspen. Our results from integrating these approaches to analyze intraspecific genetic clusters within *P. tremuloides* also obtained strong support for stable‐edge dynamics, but mixed support for trailing‐edge dynamics (*cf.* Hampe & Petit, 2005). Overall, our findings agree well with the previous genetic results of Callahan et al. (2013), but present a more nuanced picture of *P. tremuloides* evolution and diversification refining the geographical positions of genetic subdivisions, past or ongoing admixture, and putative Pleistocene refugia.

### 4.1 Quaking aspen phylogeography, admixture, and Pleistocene range shifts

Maximum‐likelihood and model‐free DAPC analyses strongly supported three genetic clusters located in coastal–Cascades (cluster 1), east‐slope Cascades–Sierra Nevada–Northern Rocky Mountains (cluster 2), and U.S. Rocky Mountains through southern Canadian (cluster 3) regions of the *P. tremuloides* range (Figures 2, 5, Figures [Link], [Link], [Link], [Link], and [Link], [Link], [Link], [Link]). These clusters were significantly differentiated based on hierarchical *F*‐statistics, and reflected in pairwise Nei's *D* distances (Fig. S10). The genetic and geographical distinctiveness of cluster 1 also supports a unique evolutionary history for the Pacific Northwest aspen populations, as suggested by taxonomists more than 100 years ago (Piper & Beattie, 1915), and hence may warrant formal taxonomic recognition. We treat this lineage as a candidate species and recommend future studies testing this hypothesis within an integrative taxonomic framework combining multiple data types (e.g., morphology, genetics; Padial, Miralles, De la Riva, & Vences, 2010).

Following the divergence of *P. tremuloides* from a common ancestor with *P. trichocarpa*, our TreeMix tree graph registered the deepest split between clusters 1 + 2 versus 3. This matches the principal genetic break reported in Callahan et al. (2013) based on microsatellite DNA markers and reveals our clusters 2 and 3 to be roughly analogous to their southwestern and northern clusters, respectively. However, Callahan et al.’s (2013) sampling in the zone between their main clusters was too sparse to clearly delineate the break beyond reference to the continental divide. We sequenced material from site POTR and two other sites near their break zone (BNF and their USF site, labeled SFRG herein) but also added samples from nearby stands in northeastern Washington, northwestern Montana, and Colorado. Probably due to our much broader sequencing of random nuclear loci from throughout the genome, piecemeal advances in geographical sampling permitted us to redefine POTR as belonging to our southwestern cluster 2 rather than northern cluster 3, and to show that populations on a diagonal from northern Montana to Colorado belong to cluster 3. Rather than strictly tracking the continental divide, the deepest genetic subdivision within *P. tremuloides* matches a set of geological and elevational barriers in the Northern to Middle Rockies, but then deviates westward from the divide (which trends south through central Colorado and western New Mexico), with the presumed barrier then correlating with cold xeric desert and shrubland habitats of the northern Great Basin and Snake River Plain.

A common Pleistocene biogeographical pattern in the Northern Hemisphere is isolation in allopatric refugia followed by postglacial dispersal and secondary contact of independent evolutionary lineages (Avise, 2000; Hewitt, 2001; Swenson & Howard, 2005). Cold desert regions of the break zone above agree well with the split between the “south‐west‐south” and “south‐west‐north” subclusters uncovered in Callahan et al. (2013), which they hypothesized to form a secondary contact zone in the eastern Great Basin. By contrast, we found individual admixture proportions within several populations (admixed individuals, left side of cluster 3 in Figure 2a) indicating a zone of admixture or secondary contact between the cluster 2 and 3 lineages, but restricted to habitats along the Rocky Mountains, and our TreeMix analyses clarified the main direction of admixture as being from cluster 3 into cluster 2 (Figure 5, Figures S17 and S18). We hypothesize that this putative area of hybridization spans from the Wasatch Range and Southern Rocky Mountains of Utah and Colorado (including sites SFRG and CSS) through the Northern Rockies (Bitterroot, Lewis, and Absaroka–Beartooth Ranges, including MON site) and into northern Idaho (POW site). Recent comparative analyses suggest that the Rocky Mountains represent a hot spot for phylogeographic breaks, hybrid zones, and contact zones across plant and animal taxa (Swenson & Howard, 2005). This area, from southern Utah through northern Montana and Idaho, also corresponds to a previously described phenotypic cline in *P. tremuloides* leaf shape, size, and tooth number (Barnes, 1975). Taken together, the intermediate SNP genotypes and clinal morphology of *P. tremuloides* in this region match theoretical and empirical expectations for “tension zones” formed by secondary contact along physical barriers, with clines maintained not by steep environmental gradients but by a balance between dispersal and local adaptation (e.g., Barton & Hewitt, 1985). A biogeographical scenario in which this zone was formed by postglacial secondary contact is supported by generally disjunct areas of niche suitability of these lineages during the LGM, followed by predicted MH and present‐day distributional overlap within the Rocky Mountains (Figure 6), which may have facilitated gene flow since ~6 ka. Several paleoecological records from the western United States and Canada suggest that expansion and contact of cluster 2 and 3 lineages, if postglacial, probably happened following ~13 ka. First, fossil pollen records from Washington (Whitlock, 1992), Alberta (Lichti‐Federovich, 1970), Wyoming (Whitlock, 1993), and southeast to Utah (Howard, 2016) do not indicate substantial (stable) percentages of *Populus* pollen in this area until ≤12–10 ka, when aspen joined pioneer forest and shrub communities that overtook preceding tundra and grassland/steppe habitats. Second, the final retreat of the Laurentide Ice Sheet opened an ice‐free Canadian corridor by ~13 ka, which was only recolonized northward by closed forests ~5 ka (Lichti‐Federovich, 1970; Pielou, 1991).

### 4.2 Stable‐ versus. trailing‐edge dynamics and isolation by distance

Testing for stable‐ versus trailing‐edge dynamics (*cf.* Hampe & Petit, 2005) is challenging, but combining ENM and genetic approaches can aid distinguishing between multiple historical scenarios or processes that could produce similar patterns (e.g., Knowles, Carstens, & Keat, 2007; Gugger et al., 2010; reviewed by Gavin et al., 2014). Based on a consensus of evidence, our geospatial and genetic results lent strong support to the stable‐edge hypothesis (H_1_) positing that “rear‐edge” *P. tremuloides* populations persisted long‐term in southwestern portions of the species range since the LGM (Callahan et al., 2013; Hampe & Petit, 2005). On the one hand, spatially explicit predictions of Pleistocene suitable areas for *P. tremuloides* and its intraspecific clusters from ENM hindcasting gave compelling indirect evidence for fully or partly stable niche suitability for clusters 1 and 2 to the southwest over LGM‐present (Figures 6 and Figure S20). Greater range stability should increase the probability of phylogeographic lineage persistence, whereas genetic lineages are more likely to collapse following secondary contact or environmental stochasticity due to range instability (e.g., Phuong, Bi, & Moritz, 2017; Singhal & Moritz, 2013). Thus, the observation that a greater number of phylogeographic lineages (2 clusters; Figure 2) occurred over LGM to present in southwestern portions of the species range also supports stable‐edge dynamics. These findings support Callahan et al.’s (2013) hypothesis that mountainous terrain promoted elevational range shifts consistent with stable‐edge dynamics in southwestern *P. tremuloides* populations during the late quaternary. Whereas this interpretation would be called into question in the face of strong longitudinal or latitudinal clines in genetic diversity in southwestern populations, we found little evidence for such clines in clusters 1 and 2 (Figure 4 and Figure S15).

The chief exception to our stable‐edge hypothesis predictions was a lack of significant isolation by distance (sensu Wright, 1943) during Mantel tests (Mantel 1967) and linear modeling of genetic and geographical distances for *P. tremuloides* clusters 1 and 2 (Fig. S12 and Table 2). This outcome is difficult to interpret, given it could indicate that assumptions of IBD tests such as migration–drift genetic equilibrium or spatial and environmental homogeneity (in ≥1–2 dimensions) are not met for a particular gene pool. We interpret nonsignificant IBD test results for clusters 1 and 2 as suggesting that a stepping‐stone or hierarchical model does not fit the data (e.g., Bohonak, 2002; Slatkin, 1993) and that a scenario of genetic drift within genetically cohesive clusters is too simplistic. If clusters 1 and 2 actually experienced stable‐edge dynamics suggested by our genetic and ENM results, then this could be explained by local subpopulations experiencing varying levels of isolation and migration. For example, this could reflect the Cascadian–Northern Rockies disjunction seen in cluster 2 subpopulations, or clusters having low *N*_e_ or migration levels near range margins could have caused *F*_ST_ to become underpredicted by an IBD model (see McRae, 2006). Still, cluster 1 exhibited greater variation in linearized *F*_ST_ than the other clusters, consistent with more ancient and genetically differentiated populations along stable edges (upper right, Fig. S12). We hypothesize that differences in the significance of IBD tests for the southwestern subpopulations between the two studies may be attributable to sampling artifacts caused by Callahan et al.’s (2013) inclusion of a highly disjunct Mexican subpopulation. Nevertheless, to obtain a better understanding of the effects of dispersal limitation in *P. tremuloides*, or potential for long‐distance dispersal disrupting IBD patterns, we recommend additional studies extending our analyses of genome‐wide SNPs to a greater density of southwestern subpopulations.

In contrast to the overall pattern of strong support for stable‐edge dynamics in the southwestern species range, we found decidedly mixed support for the trailing‐edge hypothesis (H_2_) of complete latitudinal range displacement during the LGM followed by northward postglacial expansion (Callahan et al., 2013; Hampe & Petit, 2005). Consistent with initial expectations, ENM hindcasting provided geospatial evidence for trailing‐edge dynamics overall and especially within cluster 3 (Figures 6 and Figure S20). Indeed, the inferred contraction of cluster 3 to a mid‐continental glacial refugium matches well with previous LGM–recent ENM hindcasting results (Ding et al., 2017) and paleobotanical records of *Populus* species shifting to cool mixed boreal and nonanalog forests in the same area ~18 ka during the LGM and subsequently expanding northward to higher temperate latitudes with other tree species (e.g., *Alnus* and *Abies* pollen types; Jackson et al., 2000; Williams et al., 2004; Breen et al.*,* 2012). However, contradicting expectations based on these studies, previous genetic results (Callahan et al., 2013), and theoretical predictions that populations near refugial locations should harbor greater genetic diversity (Excoffier et al., 2009; Hewitt, 1996, 2001), we failed to recover clear genetic imprints of northward postglacial range expansion for *P. tremuloides* as a whole and for cluster 3 (Figure 4 and Figure S15). These findings conflict with other studies of Pacific Northwestern plants that found reductions of genetic diversity during northward postglacial expansions in perennials and conifers (reviewed by Jaramillo‐Correa et al., 2009; Soltis et al., 1997). The lack of clinal genetic variation with latitude within *P. tremuloides* in our study could potentially have been caused by errors in genetic diversity estimation introduced in some cases by small within‐population sample sizes, by historical signals of postglacial expansion having been erased in some parts of the species range by gene flow or genetic drift, or other processes. We recommend that future studies of *P. tremuloides* phylogeography or population genetics revisit this hypothesis, particularly for cluster 3, by increasing numerical within‐population sampling and spatial sampling density.

### 4.3 Polyploidy in quaking aspen

Given whole‐genome duplications and other polyploidization events (e.g., hybridization) are common in plants (e.g., Jiao et al., 2011; Wendel, Jackson, Meyers, & Wing, 2016), an adequate understanding of plant evolution, including the analysis and interpretation of population genetic data (e.g., Meirmans et al., 2018), requires information on ploidy variation. Recent years have witnessed a surge of interest in inferring ploidy directly from high‐throughput sequencing read data obtained from fresh plant material or herbarium specimens (e.g., Gompert & Mock, 2017; Viruel et al., 2019; Weiß et al., 2018). Using these techniques, our results show that it is feasible to establish the ploidy levels of *P. tremuloides* samples from GBS reads using GMM and ML approaches implemented in nQuire (Weiß et al., 2018), which are based on modeling SNP allele frequency histograms. Tetraploids were rare enough (*n* = 2) to view them with low confidence, as they possibly reflect ploidy ambiguity, estimation errors, or sample contamination. However, we feel more confident in our inferences of triploid samples, which were from the same intermountain subpopulations or areas recognized as harboring the most triploids in previous studies (Gompert & Mock, 2017; Mock et al., 2012), and our results documented new instances of triploidy, for example, in eastern Canadian sites SFQ and HSPQ (Figure 1). Still, the overall distribution of polyploidy in our sample was minimal (14%), and instances of polyploid samples did not exert predicted effects of increasing genetic diversity in local subpopulations, based on GLM results. Clearly, deviations from expected heterozygosity patterns under polyploidy have resulted from other unknown processes, such as founder effects or genetic drift in northern populations that were recently recolonized following the LGM. Reanalyses in ADMIXTURE also showed that the presence of polyploid individuals had essentially no effect on our population structure or admixture proportion inferences (Appendix S1). Combined, these facets of our results suggest that polyploidy has likely had limited effects on our genetic inferences and hypothesis tests that were sensitive to potential fluctuations in genetic variation wrought by polyploids, including GLM cline analyses (dependent on *H*_O_, etc.) and IBD analyses (dependent on *F*_ST_ estimates) used to test predictions of the stable‐edge and trailing‐edge hypotheses. Other results based on methods that relied upon the patterns of homologous SNPs rather than their frequencies, for example, our TreeMix results, were probably not influenced by the presence of polyploids at all.

### 4.4 Biogeography of Pacific Northwest and Rocky Mountain mesic forests

The question of how disjunct mesic forest species of the Pacific Northwest came to obtain their present distributions has long fascinated biogeographers (Daubenmire, 1975; Brunsfeld et al., 2001; Brunsfeld & Sullivan, 2005). While dominated by Douglas‐fir and “cedar–hemlock” forests interspersed with xerophytic pinelands (Brunsfeld et al., 2001), this ecosystem hosts three species of *Populus*––*P. angustifolia*, *P. tremuloides*, and *P. trichocarpa* (Eckenwalder, 1996; Little, 1971). *Populus tremuloides* cluster 2 exhibits a disjunct distribution between the Cascades/Sierra Nevada ranges and the Northern Rocky Mountains, which is a common forest pattern thought to be maintained by the action of arid shrubland/steppe habitats of the Columbia Plateau as a barrier to gene flow (Brunsfeld et al., 2001). Phylogeographic structuring and past Pleistocene niche suitability patterns inferred herein suggest that *P. tremuloides* cluster 2 entered this ecosystem and obtained a disjunct mesic forest distribution in the Pleistocene, after two major historical events impacted the region: (a) Pliocene uplift of the Cascades Range and (b) xerification of the Columbia Plateau ~2 million years ago (Ma) in the early Pleistocene (Daubenmire, 1975; Brunsfeld et al., 2001). The chief reason for this is that, given Pleistocene glacial stages were much longer than relatively short ~10,000‐ to 20,000‐year interglacials, the stability of *P. tremuloides* cluster 2 suitable areas in these mountain ranges over the last glacial cycle (Figure 6) opens the possibility that the ancestral cluster 2 stock persisted in these areas during earlier glaciations over the last ~800,000 years (Lambeck, Esat, & Potter, 2002; Pielou, 1991). Nevertheless, we find limited genetic divergence between Cascade and Northern Rocky Mountain populations, as indicated by *F*_ST_, Nei's *D*, and clustering tree topologies and heatmaps of the distances (Figures 3, 5, Figures S10 and S11), suggesting that major Plio‐Pleistocene vicariant events above never sundered a continuous ancestral population. Overall, these results reject the ancient vicariance hypothesis (H_3_) but more closely resemble patterns of genetic variation expected under the inland dispersal hypothesis, our H_4_ (e.g., Brunsfeld et al., 2001). Genetic predictions of the inland dispersal hypothesis have similarly been supported by previous phylogeographic results for other forest tree species (Cartens et al., 2005; O’Connell et al., 2008).

Our phylogeographic results also clearly demonstrate that catastrophic postglacial floods that swept across the Pacific Northwestern landscape repeatedly during the Pleistocene have not played a major role in shaping population structuring within *P. tremuloides*. In contrast to initial predictions for the Missoula floods hypothesis, *P. tremuloides* from the Pacific coast and Idaho–Montana were classified by ADMIXTURE and DAPC into separate genetic clusters (Figure 2). This is despite our ENM results for the LGM, under multiple paleoclimatic scenarios, showing that suitable habitat likely existed for *P. tremuloides* across the Columbia Basin and nearby regions from LGM to present, particularly for cluster 2 (Figure 6). Moreover, if Missoula outburst flooding had transferred *P. tremuloides* from Montana to the Pacific‐coastal zone, we might expect similar patterns of admixture in these areas; however, this expectation was met by neither patterns of admixture coefficients (Figure 2) nor migration edges supported by multiple TreeMix analyses (Figures 5, Figures S17, and S18). We hypothesize that the formation and draining of postglacial lakes including Missoula did not majorly impact *P. tremuloides* population structure due to the wind‐dispersed nature and peculiar life‐history strategy of this species (e.g., clonal stands throughout many western areas of the species range). Given the evidence of an important effect of Missoula floods on cold‐tolerant freshwater fishes and mammals (e.g., Miller, Bellinger, Forsman, & Haig, 2006; Young et al., 2017, refs. therein), we suggest that aquatic plants or rodent‐dispersed tree species may be more likely to exhibit the genomic signatures of such events, and thus might be more fruitful targets for future studies of outburst flooding effects on regional plant communities.

### 4.5 Comparative biogeography of North American *Populus*

Our results yield a late Pleistocene biogeographical scenario for *P. tremuloides* starkly contrasting elements of historical biogeographical inferences for some closely related *Populus* species, while agreeing with others. For example, balsam poplar *P. balsamifera* L. is thought to have persisted in a Beringian refugium whose genetic variation was subsequently swamped by postglacial gene exchange with southern colonists (Breen et al., 2012), which possibly expanded northward from central refugium source populations (Keller et al., 2010; Levsen, Tiffin, & Olson, 2012). On the one hand, our ENM results disagree with these findings, with results from cluster‐level ENM analyses, indicating that *P. tremuloides* was probably never present in sheltered microhabitats or contiguous ice‐free areas of the Beringian land bridge refuge during the last ice age (Figure 6, except for species‐level LGM predictions), as previously suggested for *P. balsamifera*. Here, our results for *P. tremuloides* more closely resemble previous ENM hindcasting predictions for the putative sister species of *P. balsamifera*, black cottonwood *P. trichocarpa*, which Levsen et al. (2012) found to suggest patterns of southern refugia and Pacific‐coastal stability, with northward postglacial recolonization of Pacific Northwestern and Alaskan areas. Thus, ENM results for *P. tremuloides*, taken together with ENM and genetic results for *P. trichocarpa* (Levsen et al., 2012), support the hypothesis that modern‐day *Populus* populations from Alaska and the northwestern cordillera–intermountain regions of North America are postglacial in age, and we deem this the “northwestern colonization hypothesis.” On the other hand, there remain some similarities in the Pleistocene biogeography of *P. balsamifera* and *P. tremuloides*. For example, central refugium populations of *P. balsamifera* were most likely located in unglaciated regions of the intermountain west and the central North American plains (Keller et al., 2010; Levsen et al., 2012) that overlap our predicted LGM geographical distributions for *P. tremuloides* clusters 2 and 3—areas predicted based on pollen data to have been covered by boreal forest and grassland to mixed‐conifer forest LGM habitats, respectively (e.g., Jackson et al., 2000; Williams et al., 2004). This suggests that the majority of ancestral geographical populations of *P. tremuloides* probably co‐occurred, or at least broadly overlapped, in intermixed forests with *P. balsamifera* during the LGM.

Our study provides a foundation for additional research using *P. tremuloides* as a model system for elucidating processes shaping the continental‐scale patterns of geographical distributions, genetic diversity, and local adaptation of wide‐ranging forest tree species. First, additional studies of fossil pollen or genome‐scale phylogeography based on expanded geographical sampling are needed to test in further detail the northwestern colonization hypothesis outlined above, which we could not assess due to a lack of genetic samples from extreme northwestern North America, as well as other historical biogeographical scenarios. We chose to estimate tree graph relationships of *P. tremuloides* genetic clusters, in a model that adds migration edges between lineages on the tree. However, a phylogeography study of quaking aspen such as that suggested above, based on expanded sampling, would be better positioned for making statistical phylogeographical inferences of the best‐fit model describing the timing and demographic changes associated with divergences of *P. tremuloides* genetic clusters inferred herein (e.g., Gutenkunst et al., 2009; Robinson et al., 2014; Boehm et al., 2015; Menon et al., 2018). Such a study would also be able to improve on our study by testing and refining our inferences about the timing and magnitude of admixture between *P. tremuloides* clusters 2 and 3. Second, we also recommend that future studies build on our results by conducting comparative phylogeographical analyses of *Populus* and codistributed forest tree species from the Pacific Northwest and Rocky Mountain cordilleras, based on integrations of genome‐wide genetic data and ecological data similar to the methods used herein, but also employing model‐based simulation methods for comparative phylogeography (e.g., Overcast, Bagley, & Hickerson, 2017; Xue & Hickerson, 2017). Such studies would provide windows into the historical assembly of *Populus* species into North American forest communities, including insights into potential drivers of species turnover at broad continental scales and finer points of their glacial to postglacial spatial and temporal dynamics (including fluctuations in geographical distributions, e.g., precise timing of postglacial recolonization, and fluctuations in population sizes, e.g., population bottleneck and expansion events). Last, given that identifying population genomic signatures of local adaptation requires accounting for demographic history (e.g., reviewed by Rellstab, Gugerli, Eckert, Hancock, & Holderegger, 2015), our insights into putatively neutral, broadscale patterns of population genetic structure within *P. tremuloides* thus form a basis for future studies of genetic adaptation to environmental heterogeneity experienced across local populations and genetic clusters of this species.

## CONFLICT OF INTEREST

None declared.

## AUTHOR CONTRIBUTION

**Justin C. Bagley:** Conceptualization (equal); Data curation (lead); Formal analysis (lead); Methodology (lead); Project administration (lead); Writing‐original draft (lead). **Neander M. Heming:** Data curation (supporting); Formal analysis (supporting); Funding acquisition (supporting); Methodology (supporting); Writing‐review & editing (supporting). **Eliécer E. Gutiérrez:** Data curation (supporting); Formal analysis (supporting); Methodology (supporting); Writing‐review & editing (supporting). **Upendra K. Devisetty:** Data curation (supporting); Formal analysis (supporting); Writing‐review & editing (supporting). **Karen E. Mock:** Data curation (supporting); Supervision (supporting); Writing‐review & editing (supporting). **Andrew J. Eckert:** Conceptualization (equal); Funding acquisition (supporting); Methodology (supporting); Supervision (supporting); Writing‐review & editing (supporting). **Steven H. Strauss:** Conceptualization (equal); Data curation (supporting); Funding acquisition (lead); Supervision (lead); Writing‐review & editing (supporting). 

## Supporting information

Figure S1.

Figure S2.

Figure S3.

Figure S4.

Figure S5.

Figure S6.

Figure S7.

Figure S8.

Figure S9.

Figure S10.

Figure S11.

Figure S12.

Figure S13.

Figure S14.

Figure S15.

Figure S16.

Figure S17.

Figure S18.

Figure S19.

Figure S20.

Table S1.

Data S1.

Data S2.

Appendix S1.

## Data Availability

Raw sequence data are deposited in the NCBI Sequence Read Archive database (BioProject PRJNA615644). The 183‐sample genotype file with SNP calls in 012 format, filtered occurrence records and R scripts used during the ENM analysis, and the Supporting Information files are available from our Mendeley Data accession (https://doi.org/10.17632/jhkhvdgyfy.3).
