# Supplementary figures and images for: Genotyping‐by‐sequencing and ecological niche modeling illuminate phylogeography, admixture, and Pleistocene range dynamics in quaking aspen (Populus tremuloides)
Source: Ecol Evol. 2020 Apr 23;10(11):4609–29. doi: 10.1002/ece3.6214 (PMC7297775; doi:10.1002/ece3.6214)

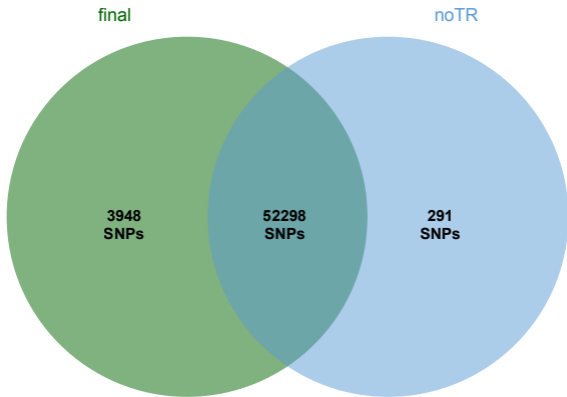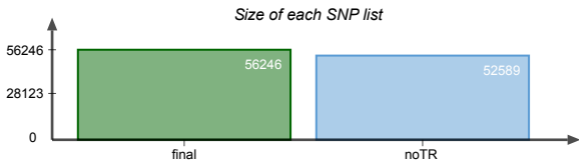

Number of elements: specific (1) or shared by the 2 SNP lists

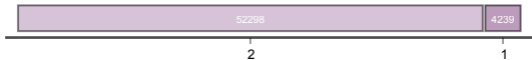

Supplement: Supplementary file 2 — Figure S2. [file ECE3-10-4609-s002.pdf]

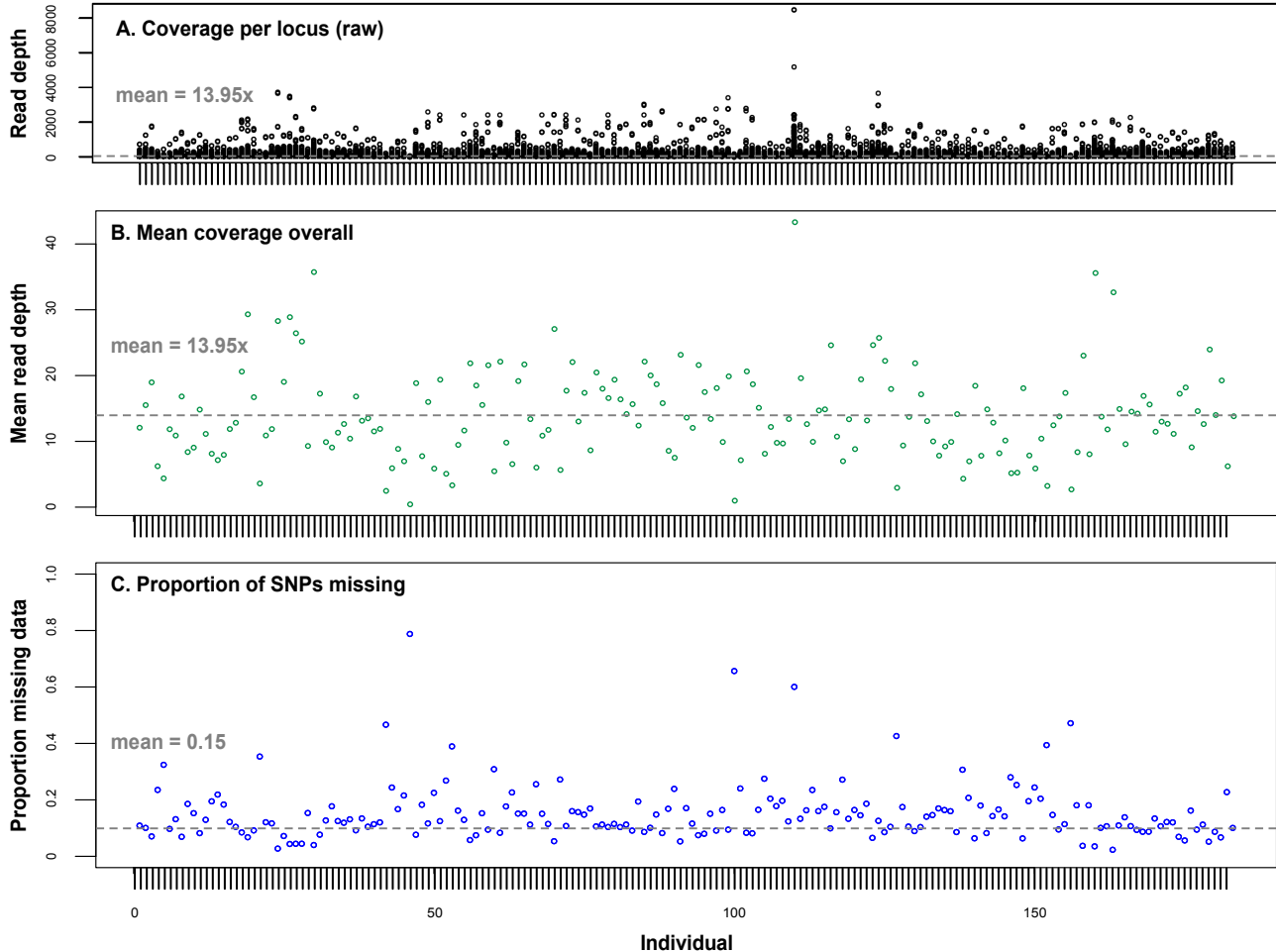

Supplement: Supplementary file 3 — Figure S3. [file ECE3-10-4609-s003.pdf]

Individual

50

100

150

Number of 2nd allele

0

1

2

5000

10000

15000

20000

25000

30000

SNP

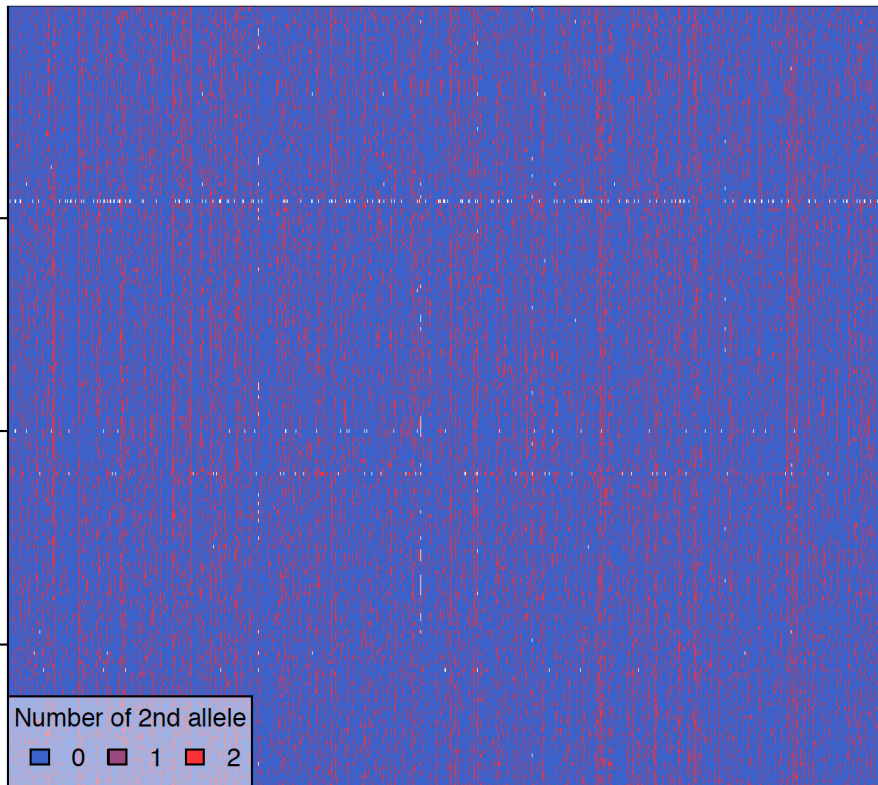

Supplement: Supplementary file 4 — Figure S4. [file ECE3-10-4609-s004.pdf]

**Cross-validation error**

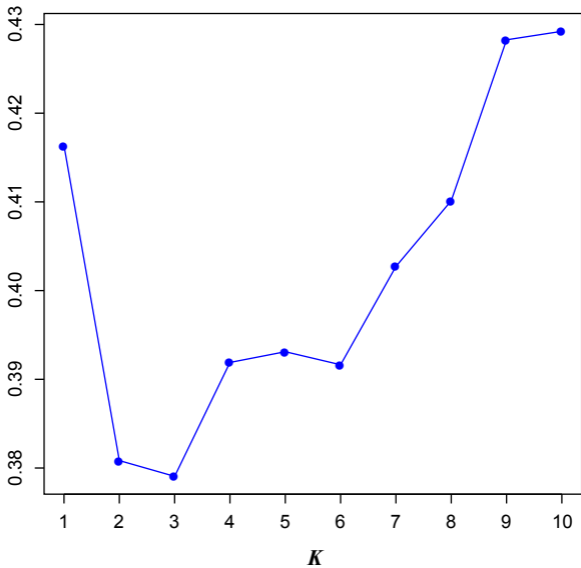

Supplement: Supplementary file 5 — Figure S5. [file ECE3-10-4609-s005.pdf]

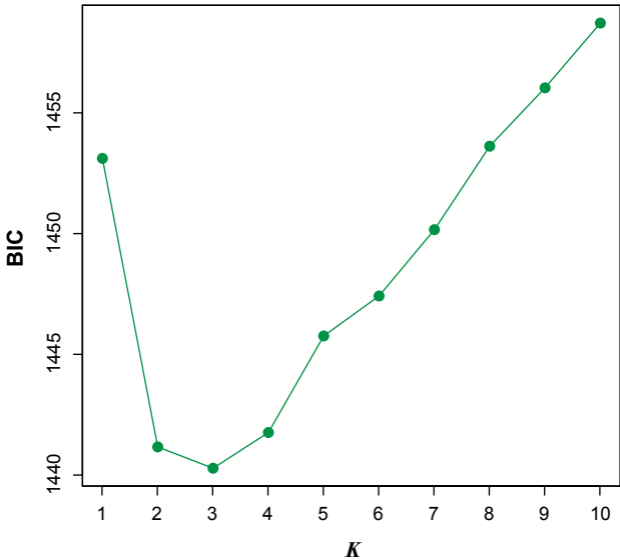

Supplement: Supplementary file 6 — Figure S6. [file ECE3-10-4609-s006.pdf]

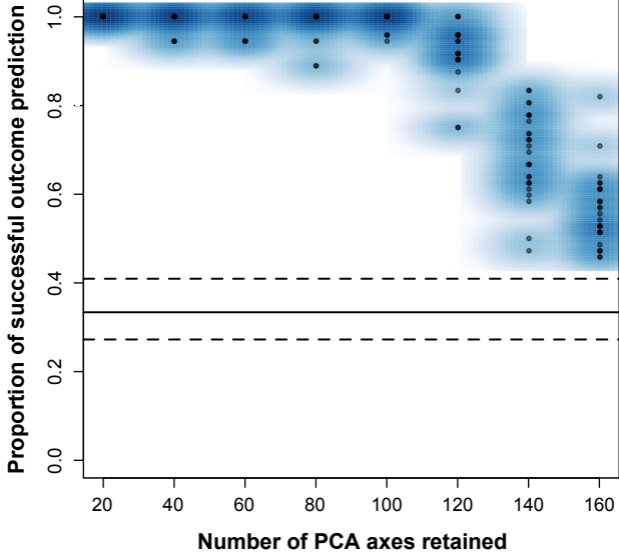

Supplement: Supplementary file 7 — Figure S7. [file ECE3-10-4609-s007.pdf]

xvalDapc DAPC (20 PCs) loadings,  $n = 34,796$  loci

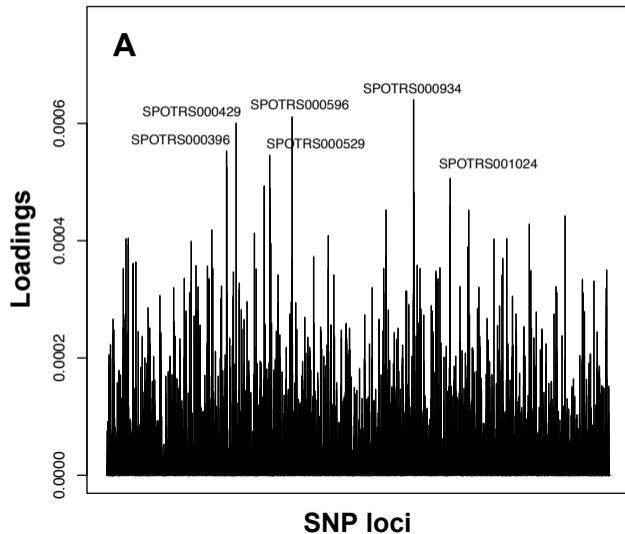

DAPC 100 (100 PCs) loadings,  $n = 34,796$  loci

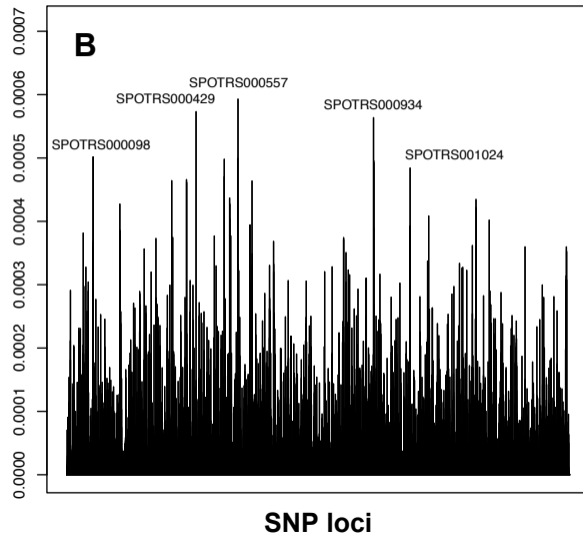

Supplement: Supplementary file 8 — Figure S8. [file ECE3-10-4609-s008.pdf]

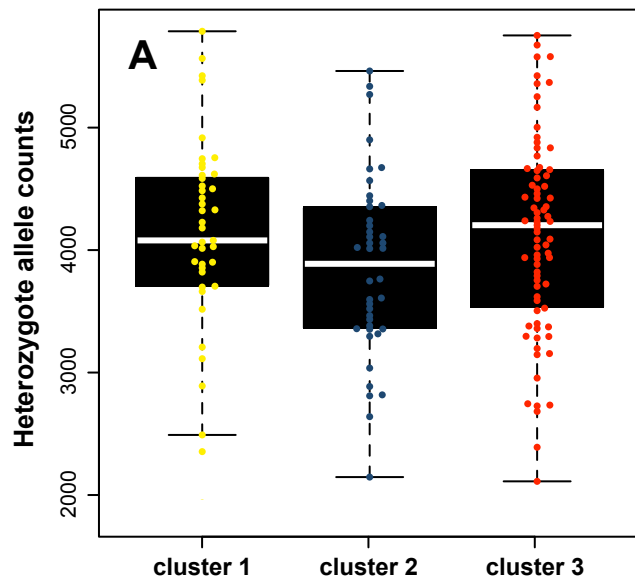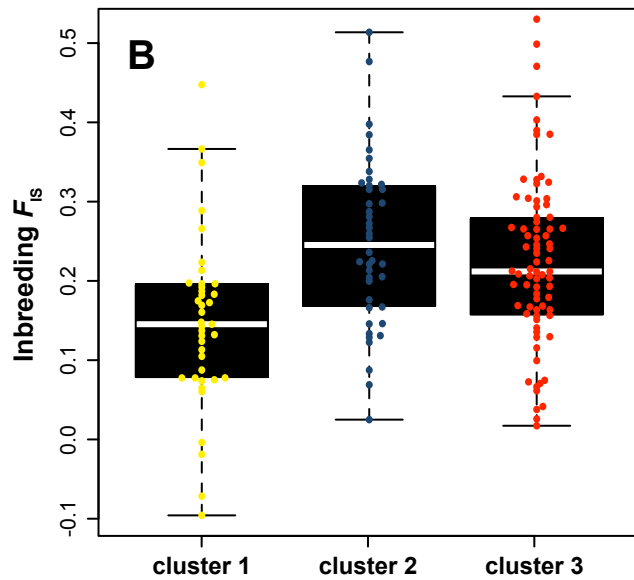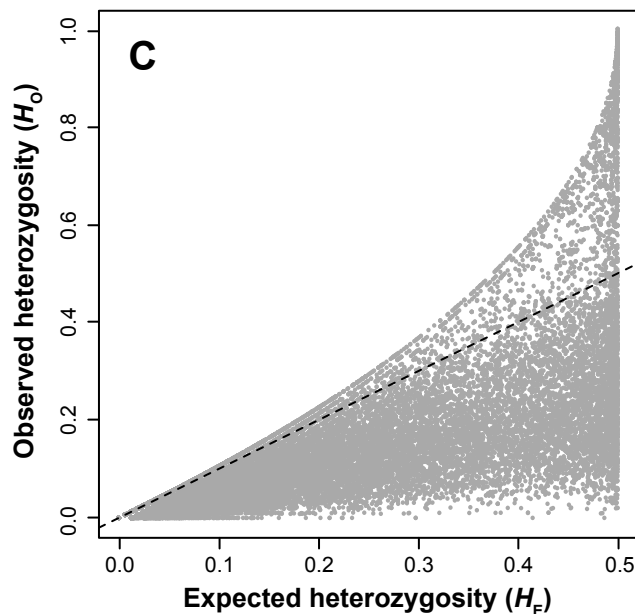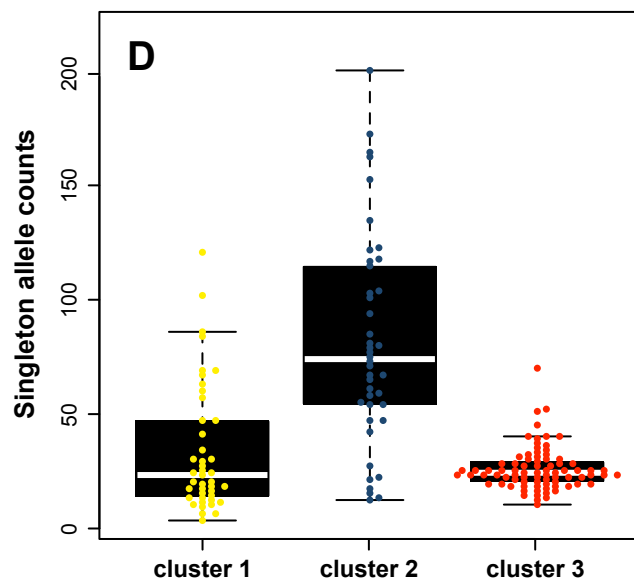

Supplement: Supplementary file 9 — Figure S9. [file ECE3-10-4609-s009.pdf]

**Color Key  
and Histogram**

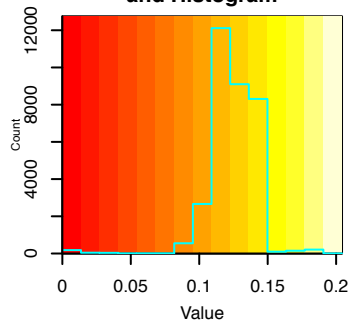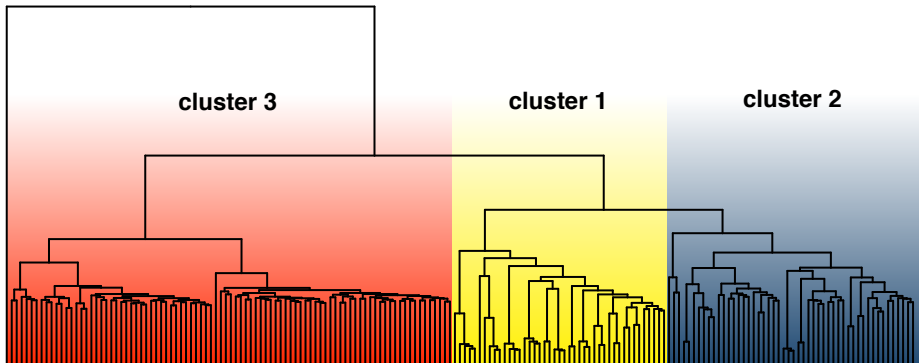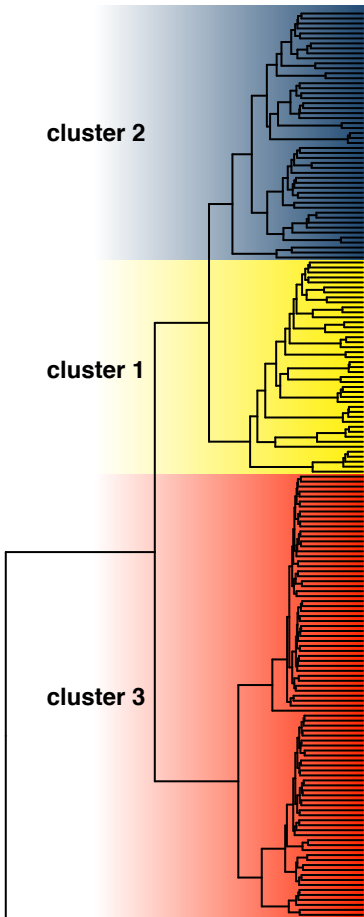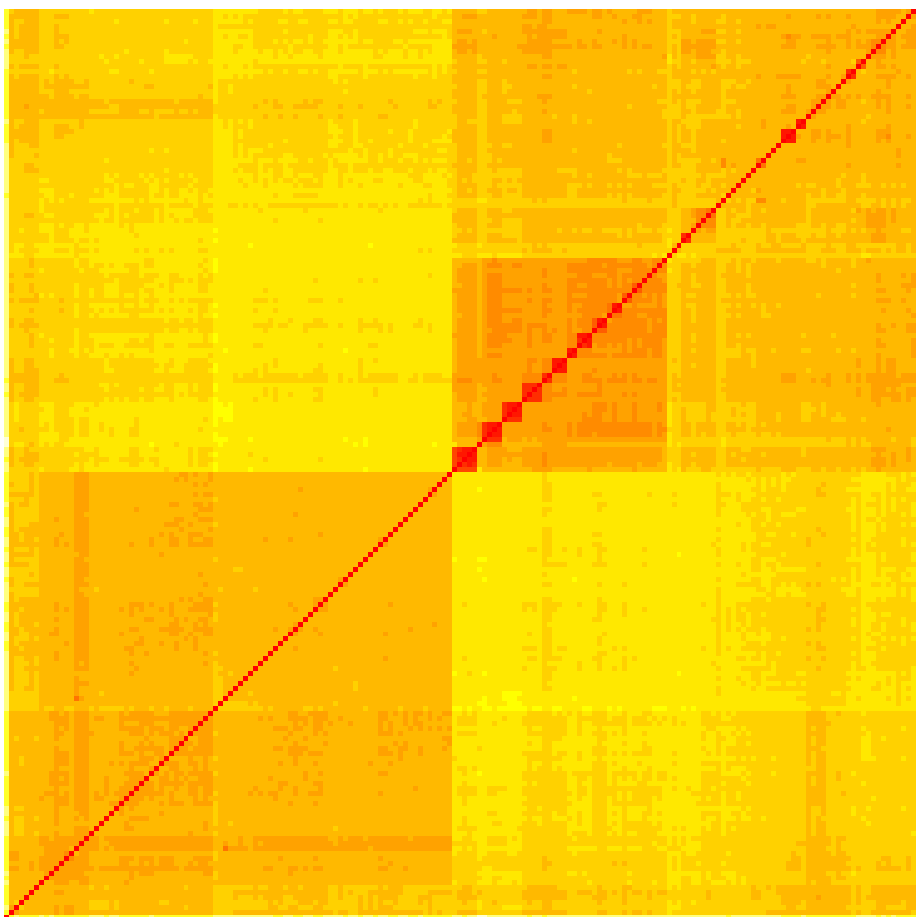

Supplement: Supplementary file 10 — Figure S10. [file ECE3-10-4609-s010.pdf]

### Color Key and Histogram

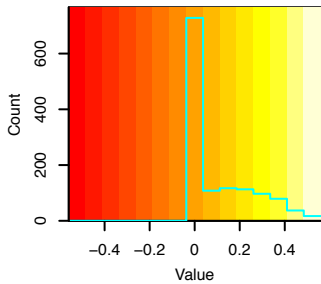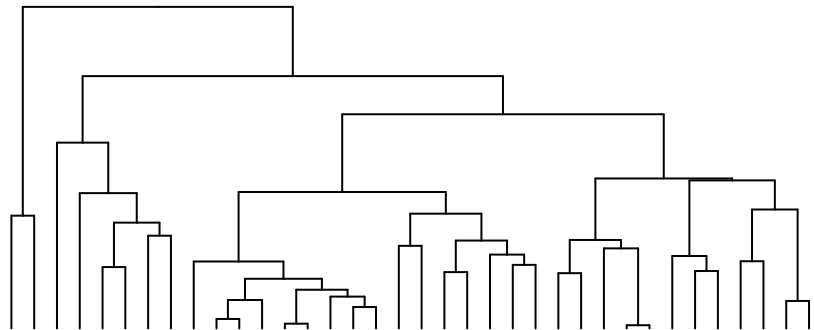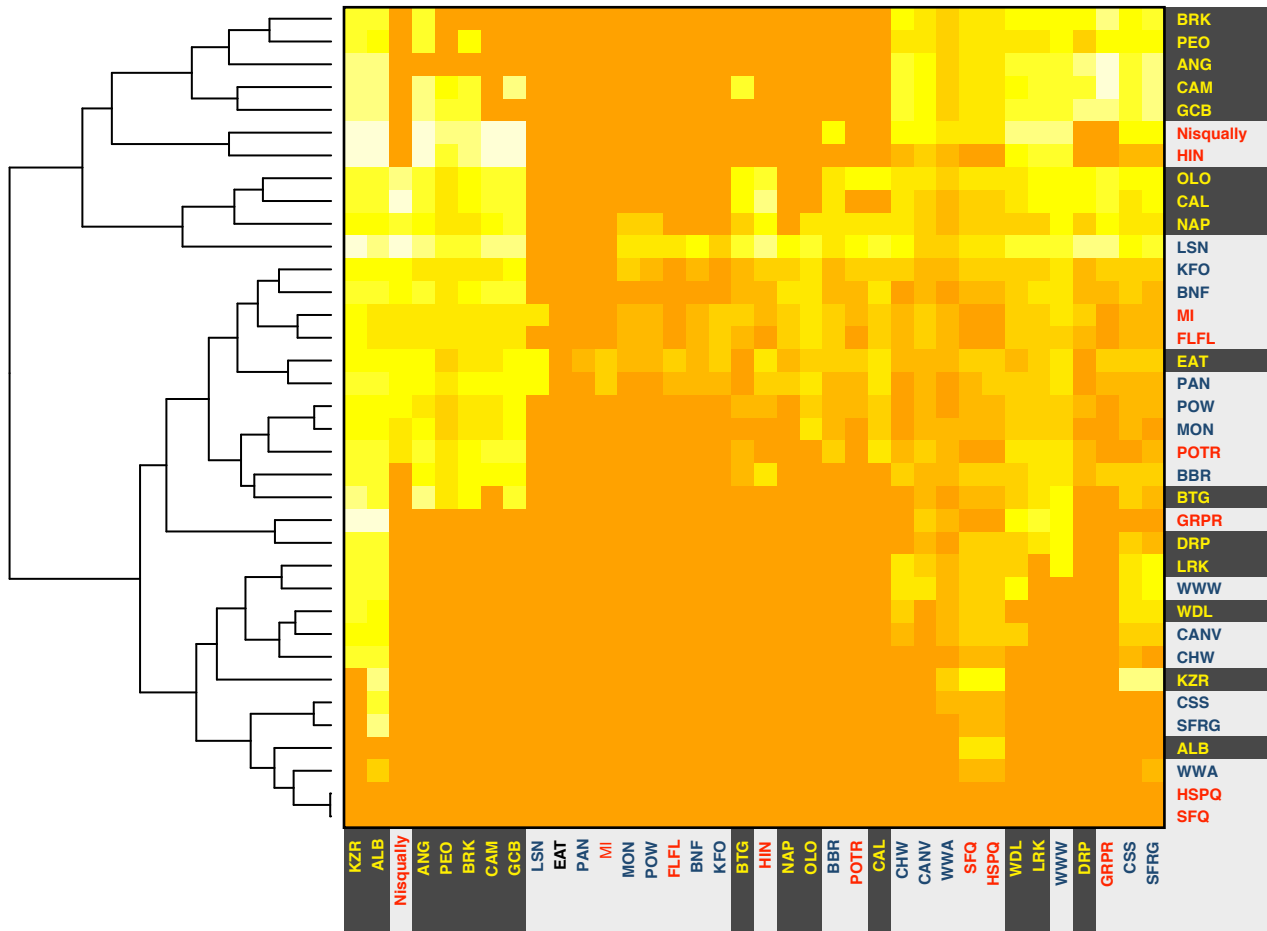

Supplement: Supplementary file 11 — Figure S11. [file ECE3-10-4609-s011.pdf]

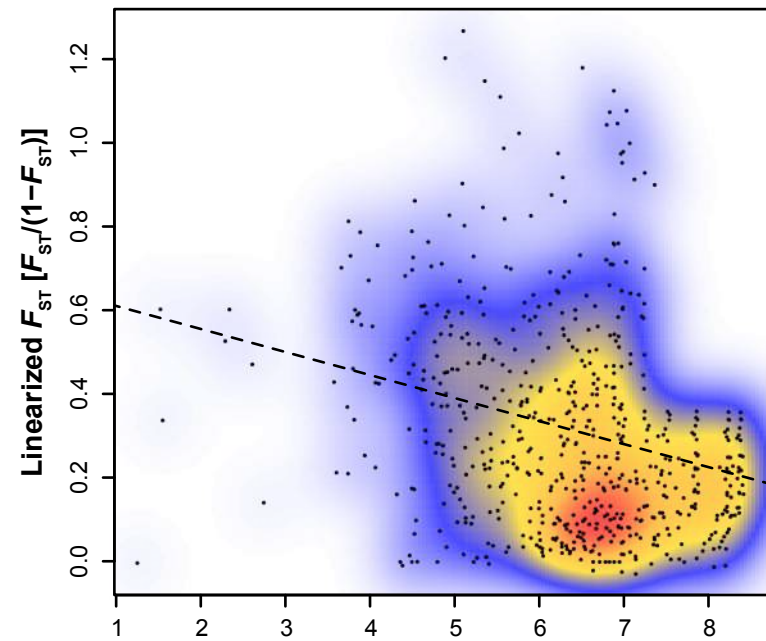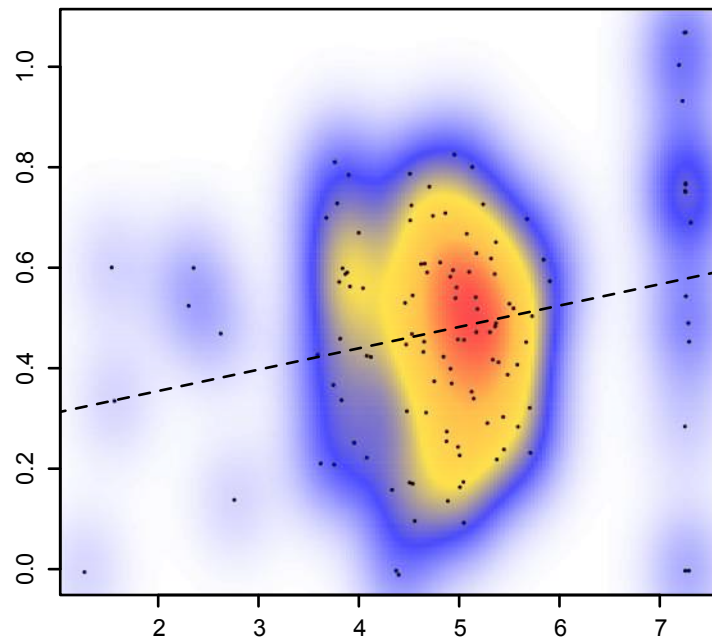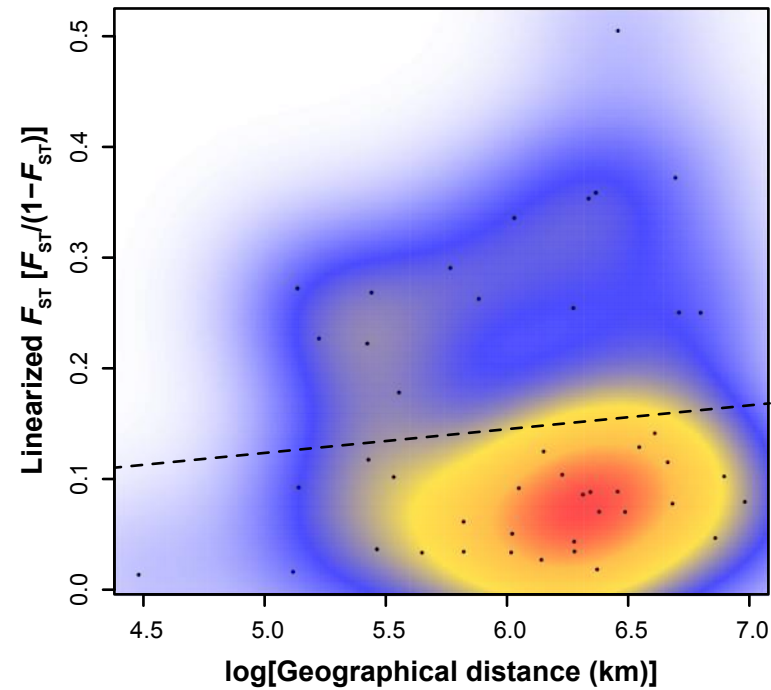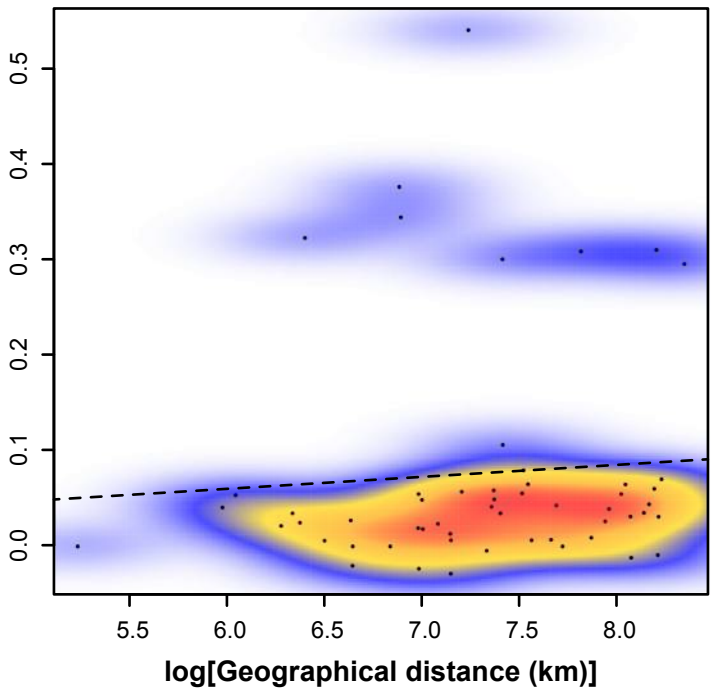

Supplement: Supplementary file 12 — Figure S12. [file ECE3-10-4609-s012.pdf]

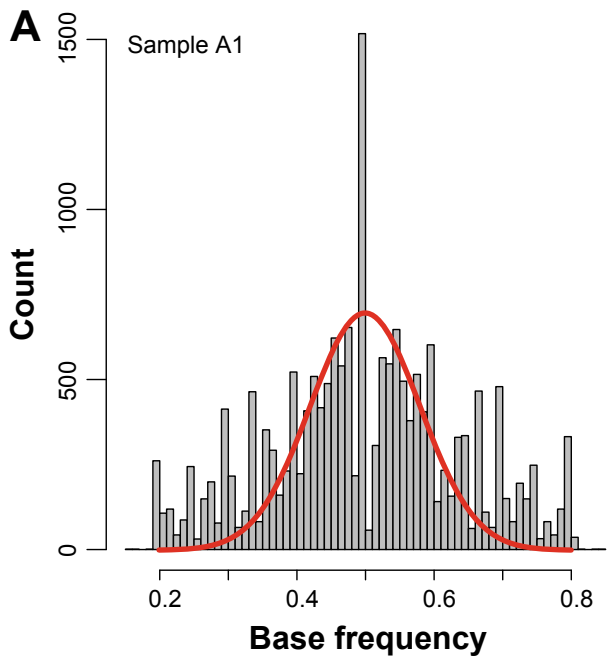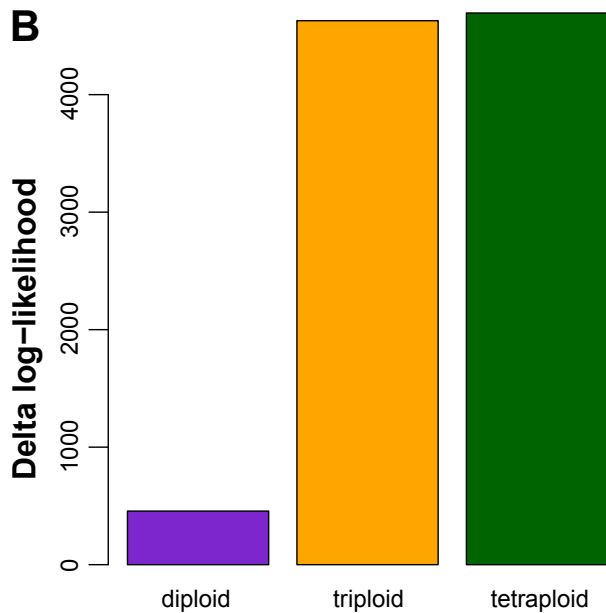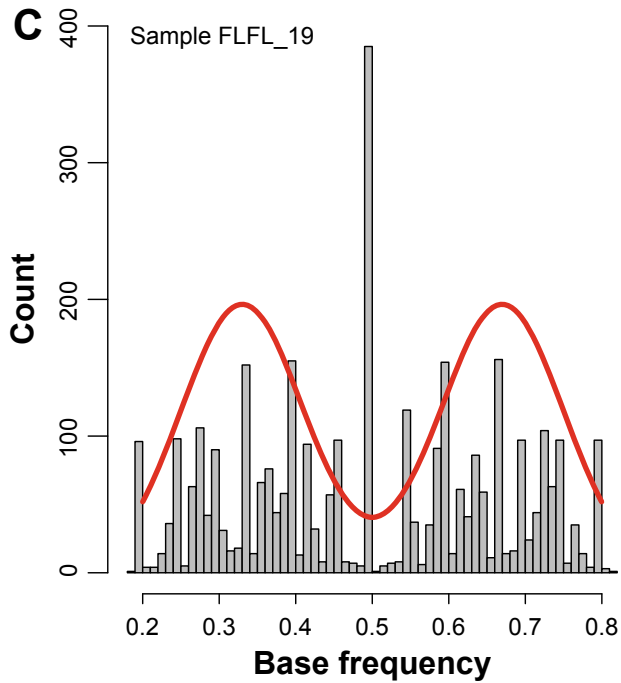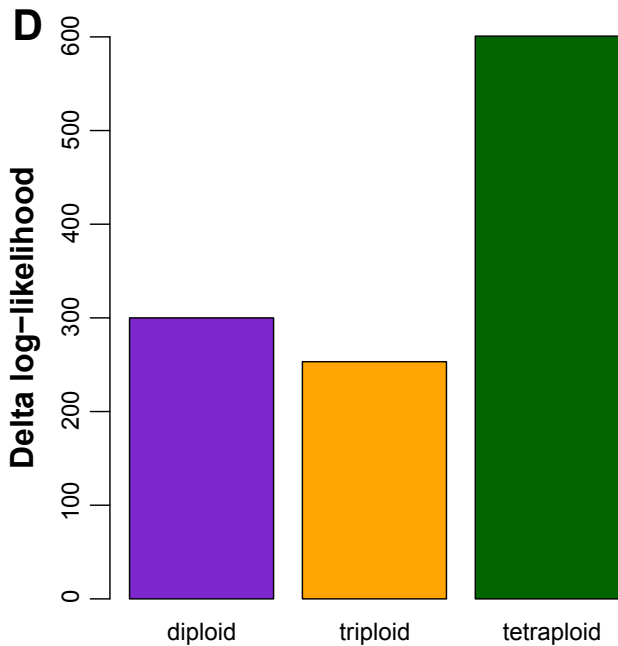

Supplement: Supplementary file 13 — Figure S13. [file ECE3-10-4609-s013.pdf]

**Polyploid count**

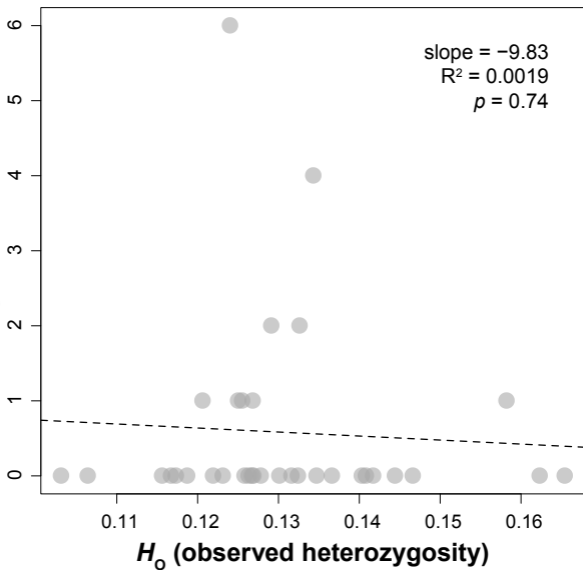

Supplement: Supplementary file 14 — Figure S14. [file ECE3-10-4609-s014.pdf]

species

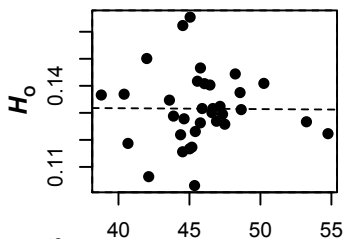

cluster 1

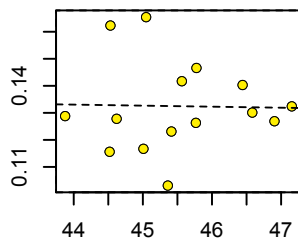

cluster 2

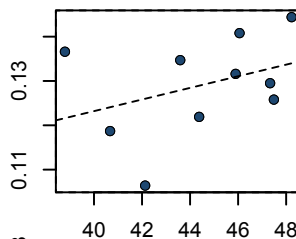

cluster 3

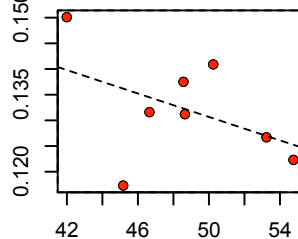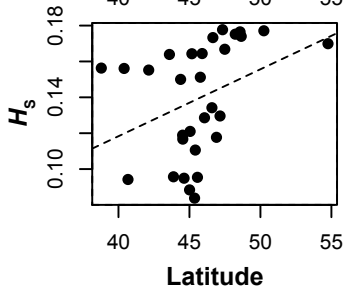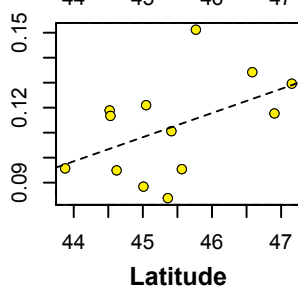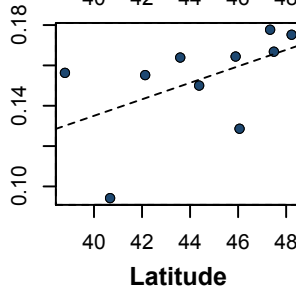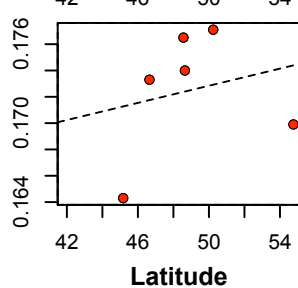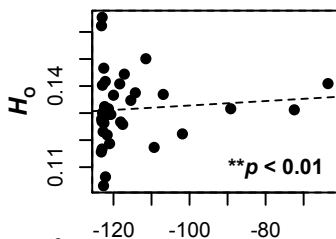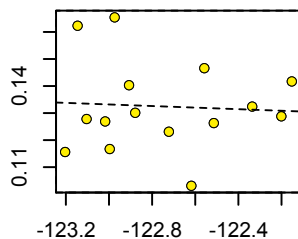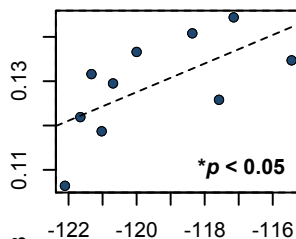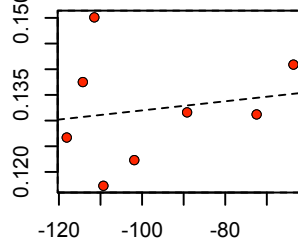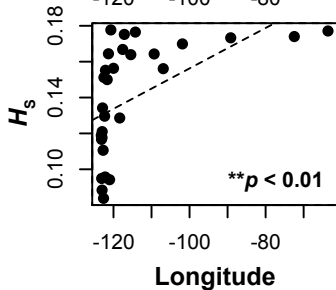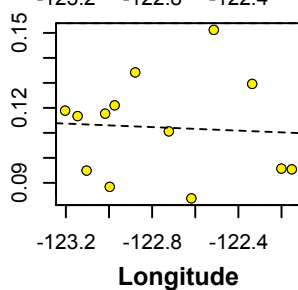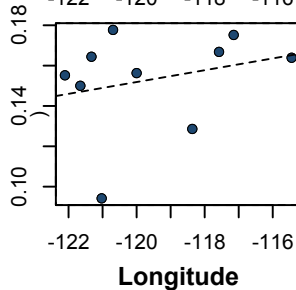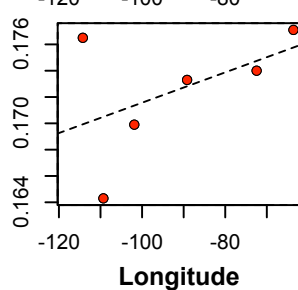

Supplement: Supplementary file 15 — Figure S15. [file ECE3-10-4609-s015.pdf]

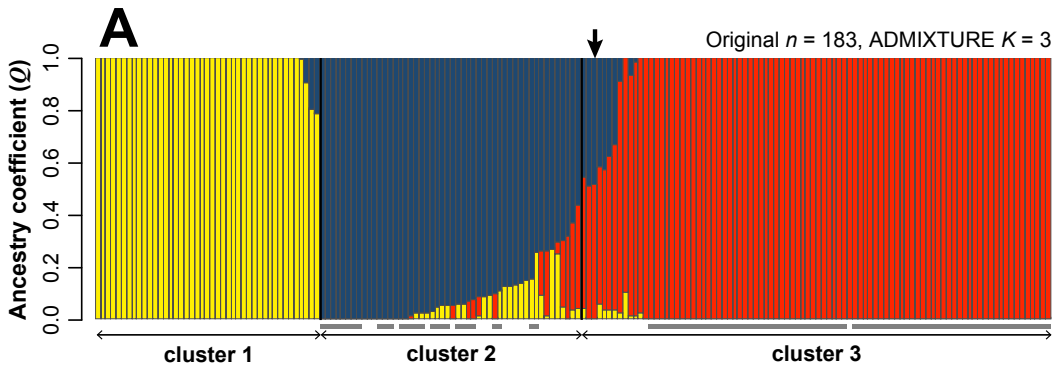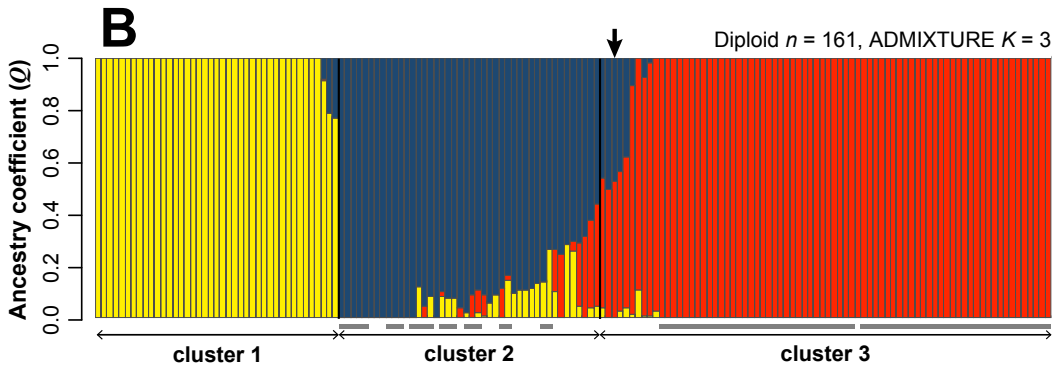

Supplement: Supplementary file 16 — Figure S16. [file ECE3-10-4609-s016.pdf]

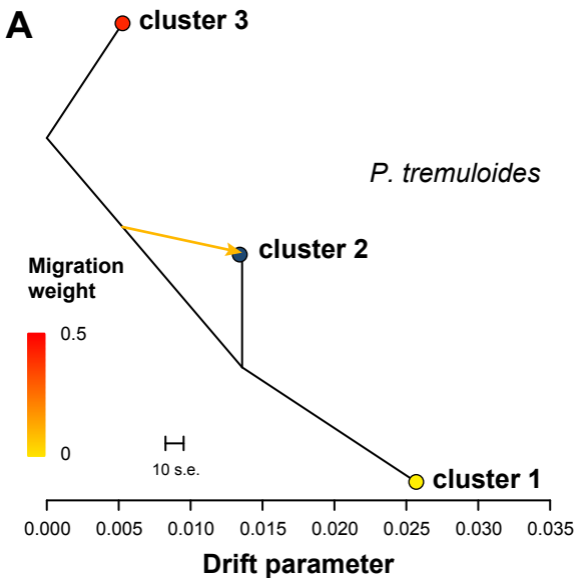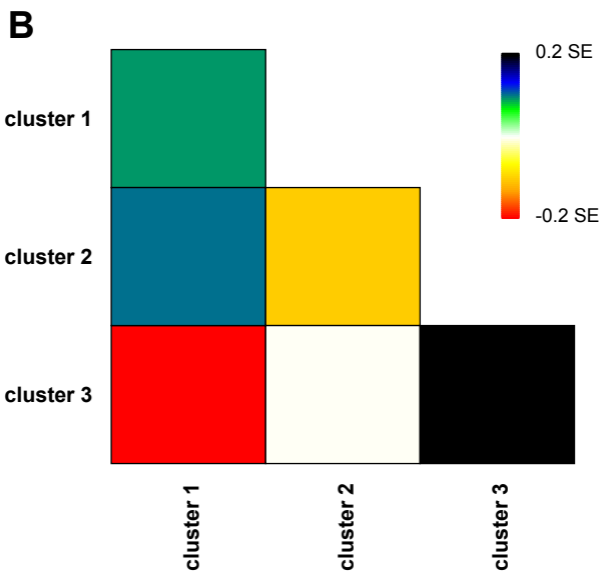

Supplement: Supplementary file 17 — Figure S17. [file ECE3-10-4609-s017.pdf]

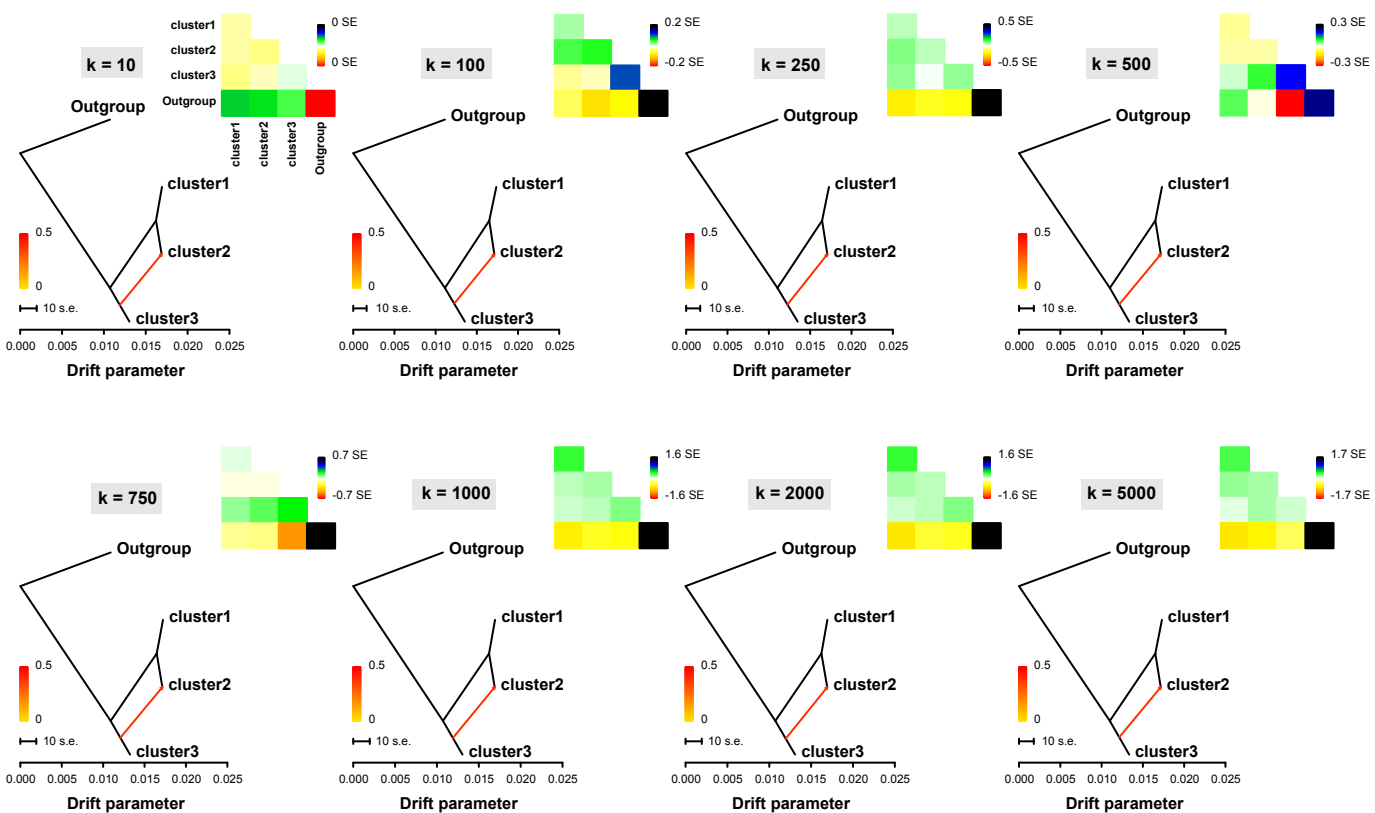

Supplement: Supplementary file 18 — Figure S18. [file ECE3-10-4609-s018.pdf]

## Calibration Area of Lineages

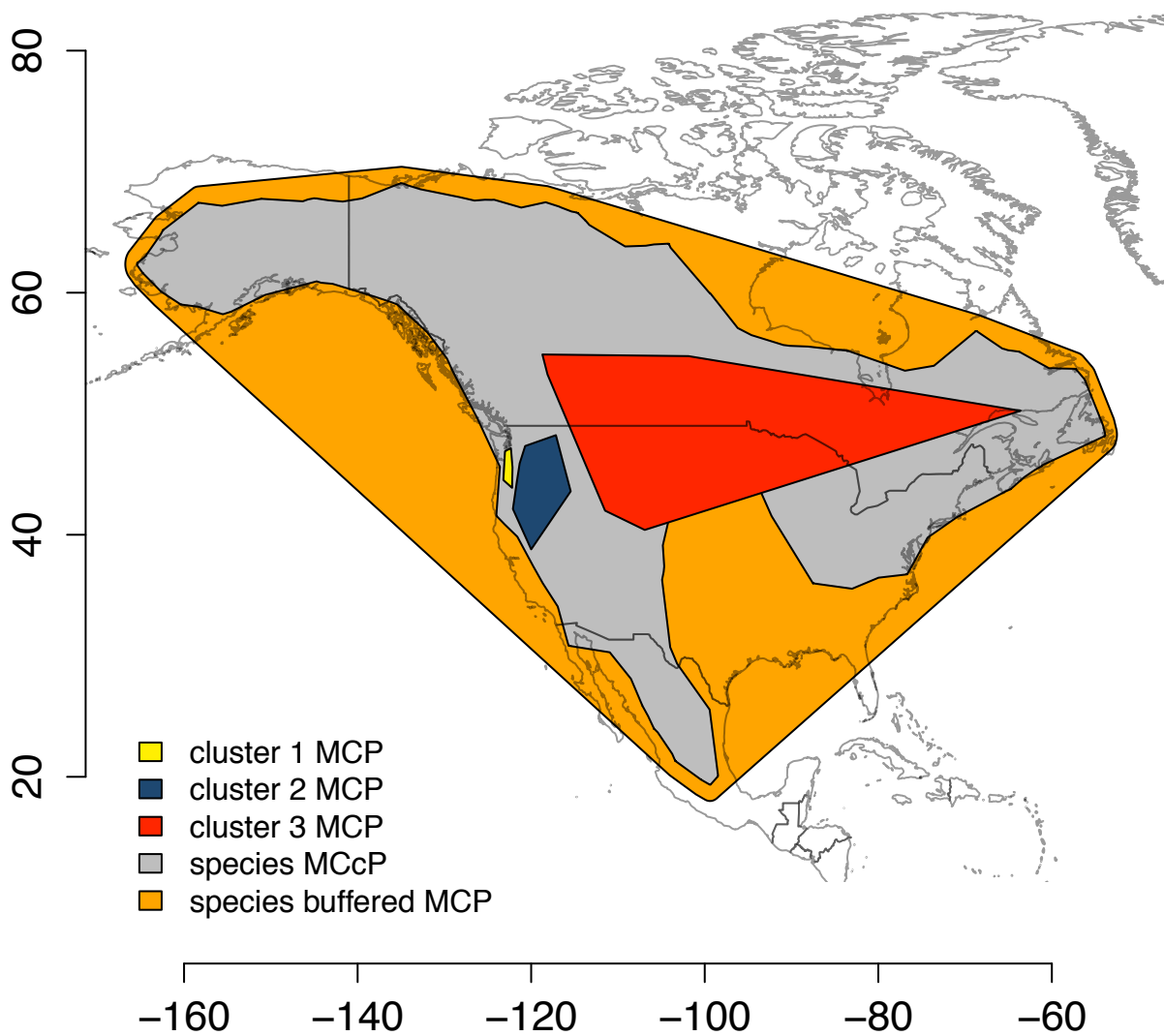

Supplement: Supplementary file 19 — Figure S19. [file ECE3-10-4609-s019.pdf]

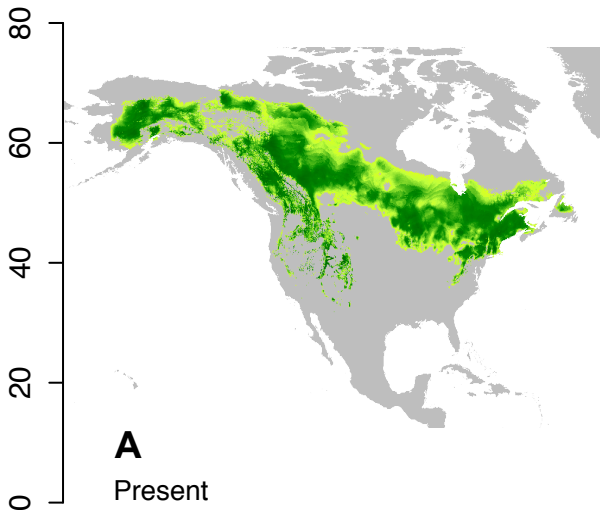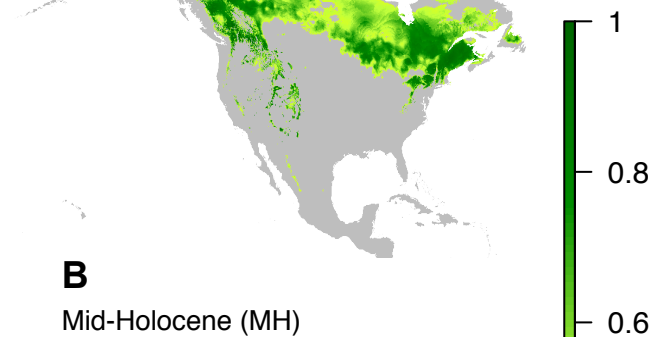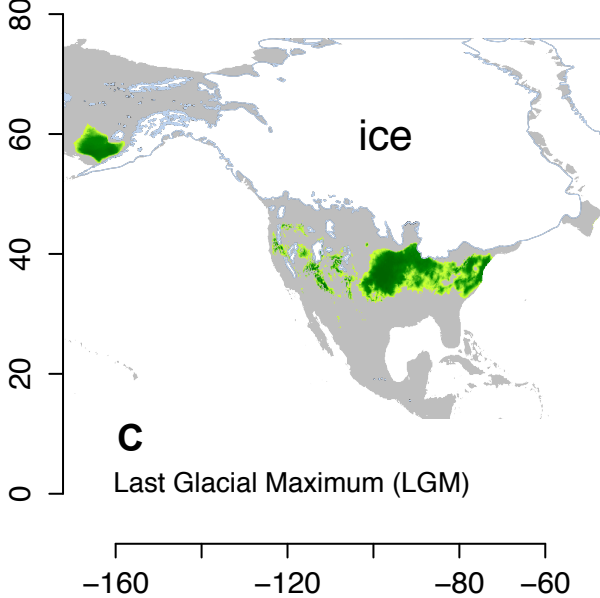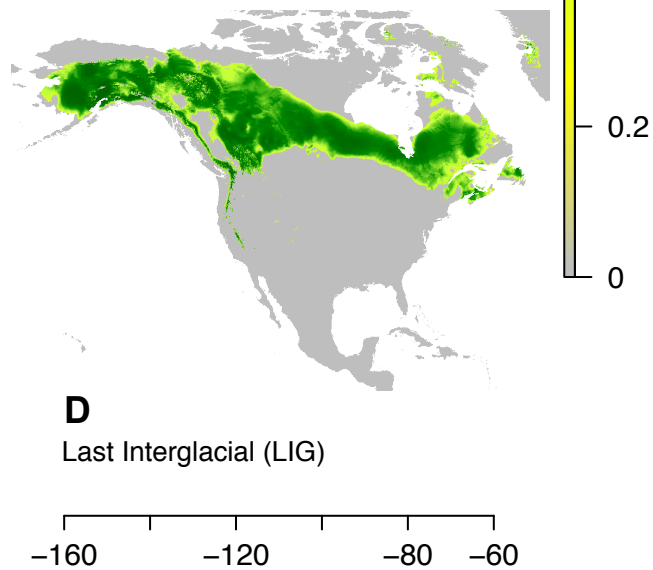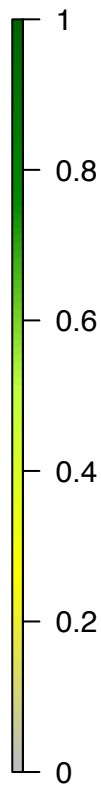

Supplement: Supplementary file 20 — Figure S20. [file ECE3-10-4609-s020.pdf]
