# Supplementary material for: Genotyping‐by‐sequencing and ecological niche modeling illuminate phylogeography, admixture, and Pleistocene range dynamics in quaking aspen (Populus tremuloides)
Source: Ecol Evol. 2020 Apr 23;10(11):4609–29. doi: 10.1002/ece3.6214 (PMC7297775; doi:10.1002/ece3.6214)
Supplement: Supplementary file 24 — Appendix S1. [file ECE3-10-4609-s024.docx]

**Appendix S1**

**Additional sampling methods.—**We obtained samples of leaves or stem cuttings from 96 *P. tremuloides* trees from 33 local sites across the species native geographical range in western North America for sequencing (Figs. 1 and 2). Samples preserved for genetic work included 1–5 individuals per site (mean = 5 individuals per site) from a Canadian site in Alberta as well as US samples from the following states (abbreviations in parentheses): Washington (WA), Oregon (OR), California (CA), Idaho (ID), Utah (UT), Montana (MT), and Colorado (CO). Additional information on the locations of the sampling sites and tissue sources of individual trees is provided in Data S1 of the Supporting Information.

After combining datasets and removing samples that failed quality checks (see main text), our final sampling included 182 *Populus tremuloides* Michx. from 36 natural populations from western and central–northern portions of the native range (Little 1971), and 1 *Populus trichocarpa* Torr. & A. Gray ex Hook. outgroup sample from Washington (not mapped; same ‘Nisqualley-1’ individual as in Schilling *et al.* 2014). A total of 79 individuals that out of the 96 samples that we sequenced for this study meet quality control criteria and were maintained in this final dataset. Due to space limitations in the main text, our presentation of sampling sites jointly with other results in Figs. 1 and 2 precluded full presentation of mapped sampling locality occurrence points and numbers which are also presented in supplementary file Data S1. To facilitate cross-referencing between these files, Fig. A1 of this Appendix provides a map of sampling sites in the final *P. tremuloides* dataset with site numbers corresponding to Data S1. The map uses the same coordinate system (WGS 1984) as Fig. 2, and it was generated in QGIS v2.14 ‘Essen’ (QGIS Development Team 2016).

**Additional bioinformatics and sequencing methods and results.—**As noted in the main text, our initial data assembly in the TASSEL-GBSv2 pipeline (Glaubitz *et al.* 2014) contained a total of 56,246 single nucleotide polymorphisms (SNPs). To evaluate potential effects of including technical replicates during the run that generated this initial SNP assembly, we re-ran TASSEL-GBSv2 on a second version of our dataset from which the 45 technical replicates from Schilling *et al.* (2014) were excluded. This was accomplished by re-running the pipeline while removing the technical replicate barcodes and IDs from the TASSEL key file. We then used the program vcf-compare (Danecek *et al.* 2011) to calculate the numbers of SNPs within, and shared between, VCF (variant call format) files resulting from the original ‘final’ TASSEL-GBSv2 run and the ‘no-technical-replicates’ (‘noTR’) run. Additionally, we determined the IDs of all SNPs in each file, as well as the intersection of SNP IDs shared between the two VCF files from these independent runs using vcftools and regex. To visualize these results, we generated a Venn diagram by inputting SNP IDs from each file into the jvenn web server (http://jvenn.toulouse.inra.fr/app/example.html; Bardou *et al.* 2014). Results are shown in Fig. A2 and demonstrate 99.4% similarity of the no-technical-replicate run SNPs and the original SNPs, suggesting that inclusion of technical replicates did not have a large influence on our SNP discovery results.

In our final set of filtered SNP variants (*n* = 34,796), we found that nucleotide patterns were biased towards A/G and C/T SNPs [A/C: 3704 (10.6%); A/G: 9847 (28.3%); A/T: 4758 (13.7%); C/G: 2597 (7.46%); C/T: 10,125 (29.1%); G/T: 3765(10.8%)]. Also, in this dataset, transitions (Ts: 19,972) outnumbered transversions (Tv: 14,824) by a ratio of 1.35.

Figure A3 shows per-individual plots of the raw and mean read depth of coverage, also referred to as simply ‘coverage’, and proportions of missing data of our final SNP dataset for *P. tremuloides*. Across individuals, mean coverage averaged 13.95× per locus (range: 1× to ­8471×; Fig. A3A) and overall (range: 1× to 43×; Fig. A3B), although the former was positively skewed, with small numbers of loci approaching read depths over 1000×. As expected, while mean coverage was the same at both levels, coverage level exhibited greater dispersion per locus (coefficient of variation, CV = 266.2) than overall (CV = 49.2). Summarizing mean values in R revealed that a total of 96% of individuals (over all loci) and 74% of SNP loci had mean coverage values ≥4×, while a total of 68% of individuals (over all loci) and 41% of SNP loci had mean coverage values ≥10×. Our final dataset was highly complete, as illustrated by the per-individual plot of the proportion of missing SNP data (i.e. SNPs in the final dataset that had ‘NA’ calls; Fig. A3C) and an individual × SNP heatmap of the data matrix contents (Fig. A4).

**Additional population genetic diversity, structure, and admixture results.—**Results from estimating pairwise *F*_ST_ and hierarchical analysis of genetic variance in the R implementation of hierfstat (Goudet 2005) are shown in Tables A1 and A2, respectively. Results from ADMIXTURE cross-validation error analysis used to select the best *K* value in the analysis of the full dataset (*n* = 183 samples) is presented in Fig. A5. Likewise, similar results used to select the optimal number of *K* genetic clusters during the *k-*means clustering step of DAPC are shown in Fig. A6. Results from DAPC cross-validation are shown in Fig. A7, and Fig. A8 shows the DAPC loading values plotted for each of 34,796 SNPs in the full dataset.

As noted in the main text, to demonstrate the limited effects of putatively admixed individuals from edge populations on our allelic patterns and genetic diversity estimates, we provide in Fig. A9 the results of several analyses conducted on a version of our full dataset that excluded these individuals, including counts of heterozygote alleles, *F*_IS_ estimates, observed versus expected heterozygosity assessment, and plotting counts of singleton alleles for the genetic clusters reported in the text. The next few appendix figures, Figs. A10–A12, present the results from additional analyses of genetic diversity and divergence between individuals and genetic clusters, as well as results from additional analyses of isolation-by-distance (IBD) mentioned in the main text.

**Additional polyploidy assessment methods and results.—**The ‘denoising’ procedure that we mentioned in the main text as being run prior to the main analyses in nQuire (Weiß *et al.* 2018) involves using nQuire’s ‘denoise’ function to downscale each BAM file with a Gaussian mixture model (GMM) that includes a uniform noise component (i.e. a GMMU model). This permits removal of baseline noise that may be present in the BAM files due to repeated elements or low genome read depth of coverage for any given individual (Weiß *et al.* 2018). The denoising procedure was necessary in our case, as read mapping steps in bwa to generate the BAM files, as well as .bin file creation steps in nQuire (where .bin files are binary files generated using the nQuire ‘create’ command), used default parameters without additional controls on read mapping quality cutoffs. When using the denoise procedure, it is also important to check the percentage of information that is maintained following denoising (*cf.* nQuire manual recommendations available at: https://github.com/clwgg/nQuire); we did perform such checks, and in doing so we ensured that denoising never left <50% of the original information content of the .bin files (per individual). As a corollary, the fact that our .bin files never lost >50% of information content also signaled that the proportion of uniform noise in our files, per individual, was always <50%.

Figure A13 provides examples of the resulting denoised read frequency histograms, as well as the corresponding delta log-likelihood scores for ploidy levels calculated in nQuire. Results are shown for one individual sample (sample A1) inferred to be diploid (Fig. A13A, B) and one individual (sample FLFL_19) inferred to be triploid (Fig. A13C, D). As shown in the barplots, the ploidy level models with the lowest difference in log-likelihood scores compared to the free model (an optimal model run without constraining ploidy level) are considered to represent the best ploidy for each sample (Weiß *et al.* 2018). We were statistically able to discriminate between ploidy models, as indicated by differences in the delta log-likelihoods.

Regarding GLMs used to test for significant positive relationships between polyploid counts and measures of heterozygosity within each *P. tremuloides* subpopulation, standard regression models were inappropriate given that count data, used for the dependent variables, are discrete and often highly skewed, and in some cases overdispersed (much greater deviance than their mean relative to expectations, e.g. under a Poisson distribution). Count data therefore are very difficult to normalize to meet assumptions of standard parametric tests and parameter estimation from ordinary least-squares regression. Such problems are often addressed using Poisson regression; however, Poisson regression does not account for overdispersion of the data, as do other related models such as the negative binomial and quasi-Poisson models. We determined the appropriate GLM parameterization for the polyploid count ~ *H*_O_ linear model using χ^2^ goodness-of-fit (GOF) tests comparing 1) Poisson link family GLMs (no overdispersion) and 2) quasi-Poisson link family GLMs (accounting for overdispersion), with 1 degree of freedom (due to only 1 predictor variable in each GLM). GOF tests used the ‘pchisq’ function in R with ‘lower.tail’ set equal to false. We also calculated deviance residuals of the Poisson model, which should be near 1 if the Poisson GLM is a good fit. If the Poisson was a poor fit, we conducted additional GOF tests comparing the fit of a Poisson GLM versus a negative binomial GLM. Statistically significant χ^2^ test results would indicate that the Poisson model is rejected at the α = 0.05 level.

Goodness-of-fit tests and other checks on our data indicated that a negative binomial GLM estimating the dispersion of the data was more appropriate than a Poisson model for the polyploid count ~ *H*_O_ GLM (*p* < 0.01). Results of the final GLM fit are presented in Fig. A14. Instead of yielding the expected positive relationship of ploidy counts with heterozygosity measures discussed in the main text, the GLM fit had a negative slope, although the underlying causal of this unpredicted negative relationship remains unknown. One potential explanation for the observed negative relationships between ploidy count and heterozygosity measures above could be related to our use of a reduced representation genome sequencing method, genotyping-by-sequencing (GBS). Specifically, the decline in heterozygosity with increased polyploid counts in *P. tremuloides* subpopulations could have resulted, in whole or in part, from triploids and tetraploids contributing additional paralogous copies of genes that our SNPs were derived from, but which were homozygous for the analogous SNP positions, contributing no new allelic variants.

In addition to the above analyses, we evaluated whether the presence of putative triploid and tetraploid *P. tremuloides* samples had an effect on our GLM tests for latitudinal or longitudinal clines in genetic diversity in R, as well as our inferences of species-wide population structure and admixture using the program ADMIXTURE. Results from re-running our GLM cline analyses using heterozygosity metrics based on putative diploids (excluding polyploids identified in nQuire above) (Fig. A15) were qualitatively nearly identical to the results of our original GLM cline analyses presented in Fig. 4, which suggests that polyploids have not exerted an undue influence on our GLM cline analyses. The main differences between these results and those of the original GLMs are that, after excluding polyploids, none of the relationships with latitude are significant, while both heterozygosity measures exhibit significant positive relationships with longitude at the species level. We also compared the results of our original ADMIXTURE analysis on the full dataset to a similar ADMIXTURE analysis of a subset of the data, from which all putative polyploids identified in nQuire were excluded. Results are shown in Fig. A16 and demonstrate that the presence of putative polyploid individuals had essentially zero effect on our ADMIXTURE analysis. Both the pattern of clusters and the ancestry coefficients for admixed individuals remain unchanged, and the main differences (Fig. A16B) are that a small number of diploids in genetic cluster 2 are inferred to have slightly higher proportions of ancestry from cluster 1, and a negligible amount of ancestry from cluster 3 is now also registered in a few of these individuals (small red box areas atop yellow box areas, middle section of Fig. A16B barplot).

**Additional tree graph analyses and admixture results.—**As noted in the main text, we here provide results from additional TreeMix analyses demonstrating the robustness of our TreeMix results to varying sampling schemes, as well as varying the block sizes used to account for linkage disequilibrium. These results are given in Figs. A17 and A18 and we refer the reader to the main text for additional discussion of them.

**Additional ecological niche modeling (ENM) methods and results.—**We modeled the existing fundamental ‘Grinnellian’ niche of *P. tremuloides* to infer the geographical distributions of climatically suitable conditions for the species both contemporaneously and in the past (see Peterson *et al.* 2011 for a review of niche theory and ENM terminology). We used ENMeval (Muscarella *et al.* 2014) to optimize regularization multiplier (RM) and feature class (FC) parameters of the MaxEnt model (Phillips *et al.* 2006), which are known to affect model complexity, overfitting, and predictions. The RM parameter is important because it penalizes overly complex models, whereas the FCs are functions of the raw environmental data (Phillips *et al.* 2006, 2017; Phillips & Dudík 2008; Elith *et al.* 2011). Also, in ENMeval, we implemented a geographic partitioning scheme. Because we transferred the present-day ENMs backwards into late Pleistocene environments by projecting the models onto paleoclimatic scenarios listed in Table 1, we used the ‘block’ partitioning scheme, which is recommended for modeling applications that requires model transferences across time periods (Muscarella *et al.* 2014).

Additionally, our ENM analyses relied on the 19 bioclimatic-environmental variables in the WorldClim 1 dataset (Hijmans *et al.* 2005). These data include monthly averages of variables derived from precipitation and temperature values initially recorded by weather stations located worldwide from 1960 to 1990 and then subsequently interpolated across weather stations. We used bioclimatic variables at a resolution of 2.5 arc-minutes, as indicated in Table 1. Any other layers that were at a higher resolution were downsampled to 2.5 arc-minutes prior to analyses; this only applied to the dataset for the LIG scenario (from Otto-Bliesner *et al*. 2006).

We conducted ENM analyses in MaxEnt at the whole-species level, as well as the level of intraspecific genetic clusters within *P. tremuloides* inferred herein and shown in Fig. 2. Species occurrence datasets often contain biases in geographic space reflective of unequal sampling effort, for example with more intense sampling near cities, research institutions, or more easily accessible areas and field sites (e.g. Reddy & Dávalos 2003). By thinning occurrences, we expected to decrease biases in geographical or environmental space reflecting unequal sampling effort, an issue that is known to adversely affect ENM analyses (see Reddy & Dávalos 2003; Boria *et al.* 2014). The final set of filtered occurrence records for *P. tremuloides* and each genetic cluster is provided in the Mendeley Data accession listed under Data Availability Statement in the main text.

When conducting ENM analysis under a hierarchical design, as in this study (species, clusters as hierarchical levels), it is important to define separate and appropriate calibration areas for each level of the analysis. The area used for model calibration at the species level was determined using methods including a minimum convex polygon (MCP) around all of the points in the final set of filtered occurrence records, using the raster R package (Hijmans 2017), and this calibration area is available in vector shapefile format (alongside that for all other calibration areas) in the ‘Calibration_Areas’ folder of our Mendeley Data accession (see Data Availability Statement). However, to generate calibration areas for each cluster, a different approach was required, and it was important to remove two kinds of areas from each cluster’s MCP. First, we removed areas included within the MCPs for the other clusters, because a given genetic cluster could be absent from the areas of closely related clusters/lineages due to competition or other unknown biotic interactions (see Anderson & Raza 2010). Second, we removed areas with *P. tremuloides* occurrence records that could pertain to unidentified lineages (due to lack of samples and genetic data from those areas). To expand, records outside the cluster MCPs cannot be reliably assigned to any of the clusters without introducing additional assumptions or biases. Thus, occurrence records within the cluster MCPs represent a geographically (and possibly environmentally) biased sample across the area potentially occupied by each lineage. This artificial bias is similar to having a barrier that prevents a species/lineage from reaching climatically suitable areas (Anderson & Raza 2010) and thus must be removed from the calibration areas (Radosavljevic & Anderson 2014; Peterson *et al.* 2011, pp. 161–162).

As a result, we delimited the whole species distribution by creating a minimum concave polygon (MCcP) around all occurrence records in the filtered set. Then, for each *P. tremuloides* genetic cluster, we excluded areas within the species MCcP but outside of the corresponding cluster MCP. The excluded areas are included as ‘MCPea’ calibration areas in our Mendeley Data accession. Under this procedure, the resulting calibration area (or ‘occurrences polygon’) for each lineage was the combination of its own MCP with areas of the species-level MCP but excluding the species-level MCcP. In supporting Fig. A19, we provide a map showing areas covered by the MCPs for the whole species (‘species buffered MCP’) and for clusters 1–3, as well as the MCcP for the species (‘species MCcP’).

The full results from projecting the present-day ENMs for *P. tremuloides* and each of its genetic clusters onto paleoenvironmental conditions from different time slices of the late Pleistocene, as modeled in data layers based on different circulation models (Table 1), is shown in Fig. 6 of the main text. While Fig. 6 has the advantage of allowing the reader to quickly compare results across the full species and each genetic cluster, over varying time slices, the mode of presentation (e.g. due to space limitations, even on full page figures) renders the overall species-level results (each panel, first column of Fig. 6) hard to see. Additionally, since Fig. 6 gives results from each time slice across multiple rows, this can make it difficult to quickly compare results from each time slice side by side. Therefore, as a complement to the ENM results presented in the main text, Fig. A20 presents projections of the present-day ENM of *P. tremuloides* onto the three late Pleistocene time slices, all based on the CCSM4 circulation model; each panel is enlarged, making the small disjunct areas of predicted suitable habitat across the Rocky Mountains and Desert Southwest much easier to see, and this mode of presentation also allows for easier comparison between time slices.

**Crowdfunding.—**As noted in the Acknowledgments section, this research was funded in part through a crowdfunding effort hosted by Experiment.com. The crowdfunding project was entitled, “The lost aspens of the Willamette Valley: Did catastrophic floods carry them from the Rockies?”, and was led by Co-PIs (co-Principal Investigators) Collin Peterson, Steve Strauss, Bill Ripple, and Rich Cronn. To acknowledge the contributions of the 68 crowdfunding donors in full, we here provide a complete list of their names, as follows:

Timothy S. Leatherman, David B. Wagner, Logan Norris, David Altman, Bruce P. Dancik and Brenda L. Laishley, Haven Baker, Bruce Chassy, Denny Luan, Terri Lomax, Jenny Kao, Ellen Watrous, David Oates, Bob Latham, Jeff Clark, Stefan Rauschen, Tina Wasem, Nicholas Wheeler, James Rinehart, Gabriela Ritokova, Sophie Duckett, Chris Wozniak, Dave Moses, Tom Adams, John Vendeland, Malory K. Peterson, Drew L. Kershen, Haiwei Lu, Kendrick Moholt, David Dalton, Al Goldner, Jim Kuhlman, Cathy L. Peterson, Linda M. Hardie, Tina Loop-Duckett, John E. Carlson, Deian Moore, Lecia Schall, Jason Holliday, Jeff Peterson, Norman Ellstrand, Jonathan Gressel, Joel Corcoran, Naomi Weidner, Laurie Simmons, Gleb Bazilevsky, Valerie Boggs, Chuck Cannon, Nick Houtman, Susan Bexton, J. Keith Gilless, Andrew Groover, Jim Border, Aaron R Leichty, David Showalter, Nancy Allen, Peggy Lemaux, Peg Silloway, Austin Strauss, Liz Swan, Oscar Jasklowski, John DeFrancisco, Mary Garrard, Tanying, Jennifer Preston Brennan, Taylor Helfand, Christina Tran, Cindy Wu, and Ryan Lower.

We gratefully acknowledge each of these individuals for their monetary support of our work, without which this paper would not have been possible.

**Appendix References**

Anderson, R.P. & Raza, A. (2010). The effect of the extent of the study region on GIS models of species geographic distributions and estimates of niche evolution: preliminary tests with montane rodents (genus *Nephelomys*) in Venezuela. *Journal of Biogeography*, *37*, 1378–1393.

Bardou, P., Mariette, J., Escudié, F., Djemiel, C., & Klopp, C. (2014). jvenn: an interactive Venn diagram viewer. *BMC Bioinformatics*, *15*(1), 293.

Boria, R. A., Olson, L. E., Goodman, S. M., & Anderson, R. P. (2014). Spatial filtering to reduce sampling bias can improve the performance of ecological niche models. *Ecological Modelling*, *275*, 73–77.

Glaubitz, J. C., Casstevens, T. M., Lu, F., Harriman, J., Elshire, R. J., Sun, Q., & Buckler, E. S. (2014). TASSEL-GBS: a high capacity genotyping by sequencing analysis pipeline. *PLoS One*, *9*(2), e90346.

Goudet, J. (2005). Hierfstat, a package for R to compute and test hierarchical *F*‐statistics. *Molecular Ecology Resources*, *5*(1), 184–186.

Hijmans, R. J. (2017). Package ‘raster’: Geographic data analysis and modeling. R package version 2.6-7. Available at: <https://CRAN.R-project.org/package=raster>.

Hijmans, R. J., Cameron, S.E., Parra, J.L., Jones, P.G., & Jarvis, A. (2005). Very high resolution interpolated climate surfaces for global land areas. *International Journal of Climatology*, *25*, 1965–1978.

Little, E. L. Jr. (Ed.) (1971). Vols. Miscellaneous Publication 1146. Digitized 1999 by US Geological Survey. US Department of Agriculture.

Muscarella, R., Galante, P. J., Soley-Guardia, M., Boria, R. A., Kass, J. M., Uriarte, M., & Anderson, R. P. (2014). ENMeval: an R package for conducting spatially independent evaluations and estimating optimal model complexity for MAXENT ecological niche models. *Methods in Ecology and Evolution*, *5*, 1198–1205.

Peterson, A., Soberón, J., Pearson, R., Anderson, R., Martínez-Meyer, E., Nakamura, M., & Araújo, M. (2011). Ecological niches and geographic distributions. Princeton University Press, Princeton, N.J.

Phillips, S. J., Anderson, R. P., & Schapire, R. E. (2006). Maximum entropy modeling of species geographic distributions. *Ecological Modelling*, *190*, 231–259.

Phillips, S. J., & Dudík, M. (2008). Modelling of species distributions with Maxent: new extensions and a comprehensive evaluation. *Ecography*, *31*, 161–175.

Phillips, S. J., Anderson, R. P., Dudík, M., Schapire, R. E., & Blair, M. E. (2017). Opening the black box: an open-source release of Maxent. *Ecography*, *40*(7), 887–893.

QGIS Development Team (2016). QGIS geographic information system. Version 2.14.3. Open Source Geospatial Foundation Project. Available at: <http://www. qgis. org/>.

R Core Team (2018). R: a language and environment for statistical computing. R Foundation for Statistical Computing, Vienna.

Radosavljevic, A. & Anderson, R.P. (2014). Making better Maxent models of species distributions: complexity, overfitting and evaluation. *Journal of Biogeography*, *41*, 629–643.

Reddy, S., & Dávalos, L. M. (2003). Geographical sampling bias and its implications for conservation priorities in Africa. *Journal of Biogeography*, *30*(11), 1719–1727.

Schilling, M. P., Wolf, P. G., Duffy, A. M., Rai, H. S., Rowe, C. A., Richardson, B. A., & Mock, K. E. (2014). Genotyping-by-sequencing for *Populus* population genomics: an assessment of genome sampling patterns and filtering approaches. *PLoS One*, *9*(4), e95292.

Weiß, C. L., Pais, M., Cano, L. M., Kamoun, S., & Burbano, H. A. (2018). nQuire: a statistical framework for ploidy estimation using next generation sequencing. *BMC Bioinformatics*, *19*(1), 122.

Weir, B. S. & Cockerham, C. C. (1984). Estimating *F*-statistics for the analysis of population structure. *Evolution*, 38, 1358–1370.

Yang, R. C. (1998). Estimating hierarchical *F*-statistics. *Evolution*, *52*, 950–956.

**Appendix Tables and Figure Captions**

**Table A1.** Pairwise *F*_ST_ estimates of genetic differentiation between *P. tremuloides* genetic clusters, and their 95% confidence intervals (CIs).

|  | **cluster 1** | **cluster 2** | **cluster 3** |
| --- | --- | --- | --- |
| **cluster 1** | – | – | – |
| **cluster 2** | 0.0613 (CIs: 0.0591– 0.0625) | – | – |
| **cluster 3** | 0.149 (CIs: 0.146–0.153) | 0.0853 (CIs: 0.0832–0.0874) | – |

Results are shown for Weir & Cockerham’s (1984) unbiased estimator.

**Table A2.** Hierarchical analysis of genetic variance among *P. tremuloides* SNPs within genetic clusters (*F*_clust/total_), within subpopulations by cluster (*F*_pop/clust_), and within individual trees relative to populations (*F*_ind/pop_) and the total variance (*F*_ind/total_). Ranges given in parentheses are 95% confidence intervals.

|  | ***F*_clust/total_** | ***F*_pop/clust_** | ***F*_pop/total_** | ***F*_ind/total_** |
| --- | --- | --- | --- | --- |
| **Overall** | 0.092 (CIs: 0.090–0.094) | 0.089 (CIs: 0.088–0.091) | 0.173 (CIs: 0.170–0.176) | 0.326 (CIs: 0.319–0.333) |

Results are shown for hierarchical *F*-statistics (Yang 1998) as computed in hierfstat (Goudet 2005) based on 100 bootstrap pseudoreplicates.

**Figure A1.** Map of geographical sampling sites for sequenced *P. tremuloides* in the final dataset. Map is similar to Fig. 2, except sampling sites (black dots) are numbered to match Data S1 of the Supporting Information. The extent of continental ice sheets during the Last Glacial Maximum (LGM) is shown with a solid light blue line. State abbreviations follow the main text and Fig. 2.

**Figure A2.** Venn diagram describing the patterns and intersection of SNPs resulting from two independent runs of the TASSEL-GBSv2 pipeline (Glaubitz *et al.* 2014) on our final dataset: the original run (‘final’) and a run excluding technical replicates (‘noTR’). Results were generated from SNP lists in VCF files from each run.

**Figure A3.** Per-individual plots of read depth of coverage and proportion missing data for the final aspen SNP dataset. Coverage results are shown as means (circles) plotted per locus (A) and overall, calculated across mean values for each locus (B), per individual (indexed left-to-right, from 1–183). Missing SNP data proportions are plotted to the same individual index (C).

**Figure A4.** Heat map of the final SNP data matrix, with cells colored by minor allele count. Individuals are indexed top-to-bottom along the *y-*axis in the same order that individuals were arranged left-to-right in Fig. A2, and white cells indicate missing data (‘NA’ calls).

**Figure A5.** Plot of ADMIXTURE (Alexander *et al.* 2009) cross-validation error versus *K*, showing that *K* = 3 is the best fit for our final aspen dataset of 34,796 SNPs (following Alexander & Lange 2011).

**Figure A6.** Plot of Bayesian information criterion (BIC) scores for *k*-means clustering solutions over a range of *K*, from the first step of DAPC, with *K* = 3 being the best solution.

**Figure A7.** Results of DAPC cross-validation in R establishing that the appropriate number of principal components to retain ranges from 20–100 with similarly high (>90%) prediction success.

**Figure A8.** DAPC loading values plotted for all 34,796 SNPs, with SNP name labels beside the SNPs with the highest loadings.

**Figure A9.** Genetic patterns of heterozygote and singleton alleles within and among *P. tremuloides* genetic clusters, calculated while excluding putatively admixed edge populations (*Q*_max_ < 0.75). Results are analogous to corresponding panels of Fig. 3, thus see Fig. 3 caption for additional details.

**Figure A10.** Heatmap of interindividual Nei’s *D* estimates, reordered by row and column means, and flanked by dendrograms of the values. Color key and histogram at top left show the distribution of mean *D* values, and sample group colors match the ADMIXTURE clusters in Fig. 2.

**Figure A11.** Heatmap of unordered interpopulation *F*_ST_ estimates flanked by clustering dendrograms from the distances. Color key and histogram at top left show the distribution of mean *F*_ST_ values. Tip label colors match ADMIXTURE clusters in Fig. 2.

**Figure A12.** Results of isolation by distance tests based on GLM analyses of linearized *F*_ST_ versus log[geographic distance (km)] of *P. tremuloides* (A) and its genetic clusters 1 (B), 2 (C), and 3 (D) from ADMIXTURE (Fig. 2).

**Figure A13.** nQuire results for one *P. tremuloides* sample (sample A1) inferred to be diploid (A, B) and one sample (sample FLFL_19) inferred to be triploid (C, D). Read frequency histograms of biallelic SNPs and delta log-likelihood scores for different ploidy models (diploid, triploid, tetraploid) are shown from analyses of the denoised .bin file for each sample. Empirical base frequencies are compared against approximate hypothetical uni- and bi-modal distributions of frequencies expected for diploids and triploids, plotted as red lines. These results illustrate the ability of our approach using denoised .bin files to statistically discriminate among ploidy levels.

**Figure A14.** Results of generalized linear model (GLM) analysis testing for a relationship between polyploid *P. tremuloides* sample counts and observed heterozygosity (*H*_O_) as a measure of genetic diversity. Results are presented for a negative binomial GLM, and the R^2^ value given is the Cox and Snell pseudo-R^2^ estimated using the ‘nagelkerke’ R function.

**Figure A15.** Latitudinal and longitudinal clines in SNP genetic diversity based on analyses of putative diploid *P. tremuloides*. Linear relationships of observed heterozygosity (*H*_O_) and gene diversity (*H*_S_) with latitude and longitude are shown for the species as a whole, and for each intraspecific genetic cluster. Each dot represents a subpopulation and is drawn to match the colors of genetic clusters defined in Fig. 2. Regression lines are drawn from fitted values of GLMs, and in cases of significant relationships, *p*-values are given in the lower right of the plot. Results in this figure are directly comparable to those in Fig. 4.

**Figure A16.** Comparison of results from the best ADMIXTURE model (in both cases, for *K =* 3) from the original analysis of the full dataset (A) versus an analysis of the same dataset, after excluding individuals identified as putative polyploids by nQuire (B). Individuals are in the same order from left to right in both barplots, formatting and genetic cluster colors match that of Fig. 2, and the vertical arrow identifies the outgroup individual. The 104 individual samples added to this study in the raw dataset from Schilling *et al.* (2014) are indicated by gray horizontal bars under the plot.

**Figure A17.** Unrooted maximum-likelihood (ML) tree topology from the ingroup-only TreeMix analysis allowing a single migration event (A), and residual plot of the graph (B). Scale bars and legends same as in Fig. 5.

**Figure A18.** Results of TreeMix analyses on the full dataset conducted over varying levels of *k* block sizes (10 to 5000 bp) accounting for linkage disequilibrium. Scale bars, legends, and residual plots follow a similar format to that in Figs. 5 and A17.

**Figure A19.** Map of calibration areas for lineages analyzed using ecological niche modeling in this study. Different polygons were created as described in the main text and this appendix for *P. tremuloides* and each of the ADMIXTURE-inferred intraspecific genetic clusters (clusters 1–3) shown in Fig. 2.

**Figure A20.** Projection of the final present-day ecological niche model of *P. tremuloides* onto three late Pleistocene climatic scenarios. The ENM was built with MaxEnt and bioclimatic variables obtained from WorldClim v1, and the three Pleistocene time-slices (B–D) correspond to scenarios described in the Fig. 6 caption and Table 1. Model projections show continuous suitability values obtained using the cloglog format of MaxEnt after the application of a 10^th^-percentile threshold. Extent of LGM ice sheets is indicated in white.
