# Supplementary material for: Genotyping‐by‐sequencing and ecological niche modeling illuminate phylogeography, admixture, and Pleistocene range dynamics in quaking aspen (Populus tremuloides)
Source: Ecol Evol. 2020 Apr 23;10(11):4609–29. doi: 10.1002/ece3.6214 (PMC7297775; doi:10.1002/ece3.6214)
Supplement: Supplementary file 1 — Figure S1. [file ECE3-10-4609-s001.pdf]

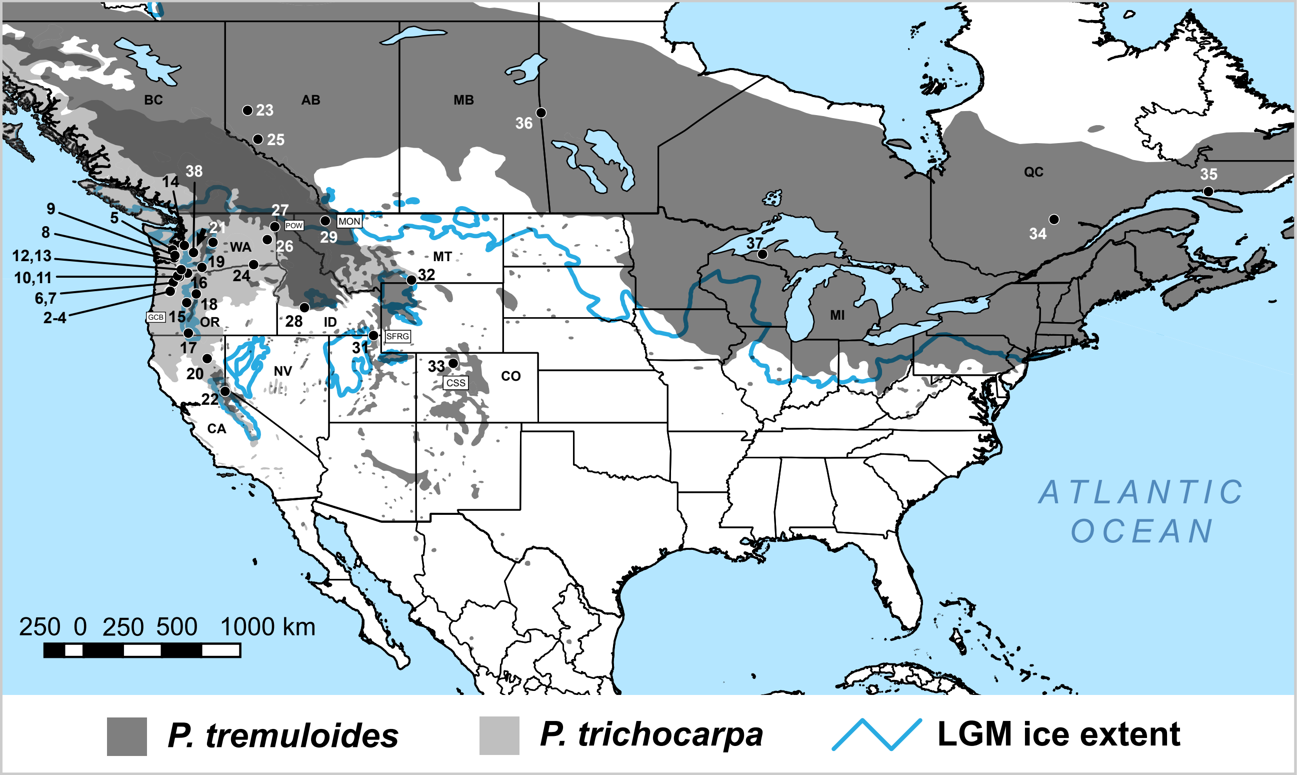

BC
AB
MB
QC
WA
MT
ID
OR
NV
CA
CO
MI
23
25
36
35
34
37
38
14
9
5
8
12,13
10,11
6,7
2-4
27
POW
MON
29
21
26
19
24
16
18
GCB
15
28
17
20
22
31
SFRG
32
33
CSS
ATLANTIC OCEAN
250 0 250 500 1000 km
P. tremuloides
P. trichocarpa
LGM ice extent
